# Empathy: Assessment Instruments and Psychometric Quality – A Systematic Literature Review With a Meta-Analysis of the Past Ten Years

**DOI:** 10.3389/fpsyg.2021.781346

**Published:** 2021-11-24

**Authors:** Felipe Fernandes de Lima, Flávia de Lima Osório

**Affiliations:** ^1^Ribeirão Preto Medical School, University of São Paulo, São Paulo, Brazil; ^2^National Institute for Science and Technology (INCT-TM, CNPq), Brasília, Brazil

**Keywords:** empathy, psychometrics, validity, reliability, instruments

## Abstract

**Objective:** To verify the psychometric qualities and adequacy of the instruments available in the literature from 2009 to 2019 to assess empathy in the general population.

**Methods:** The following databases were searched: PubMed, PsycInfo, Web of Science, Scielo, and LILACS using the keywords “empathy” AND “valid^∗^” OR “reliability” OR “psychometr^∗^.” A qualitative synthesis was performed with the findings, and meta-analytic measures were used for reliability and convergent validity.

**Results:** Fifty studies were assessed, which comprised 23 assessment instruments. Of these, 13 proposed new instruments, 18 investigated the psychometric properties of instruments previously developed, and 19 reported cross-cultural adaptations. The Empathy Quotient, Interpersonal Reactivity Index, and Questionnaire of Cognitive and Affective Empathy were the instruments most frequently addressed. They presented good meta-analytic indicators of internal consistency [reliability, generalization meta-analyses (Cronbach’s alpha): 0.61 to 0.86], but weak evidence of validity [weak structural validity; low to moderate convergent validity (0.27 to 0.45)]. Few studies analyzed standardization, prediction, or responsiveness for the new and old instruments. The new instruments proposed few innovations, and their psychometric properties did not improve. In general, cross-cultural studies reported adequate adaptation processes and equivalent psychometric indicators, though there was a lack of studies addressing cultural invariance.

**Conclusion:** Despite the diversity of instruments assessing empathy and the many associated psychometric studies, there remain limitations, especially in terms of validity. Thus far, we cannot yet nominate a gold-standard instrument.

## Introduction

There is growing consensus among researchers concerning empathy being a multidimensional phenomenon in recent years, which necessarily includes cognitive and emotional components (Davis, 2018). Reniers et al. (2011), for instance, consider that empathy comprises both an understanding of other peoples’ experiences (cognitive empathy) and an ability to feel their emotional experiences (affective empathy) indirectly. Baron-Cohen (2003, 2004) considers that empathy is the ability to identify what other people are thinking and feeling (cognitive empathy) and to respond to these mental states with appropriate emotions (affective empathy), enabling individuals to understand other peoples’ intentions, anticipate their behavior and experience the emotions that arise from this contact with people. Hence, empathy enables effective interaction in the social world.

According to Kim and Lee (2010), empathy can be assessed by self-report instruments/scales and observed through most psychological constructs. Moya-Albiol et al. (2010) stress that new strategies have been recently proposed to approach this construct from an ecological perspective, such as computational tasks with emotional stimuli.

The literature presents instruments to assess an individual’s ability to provide empathic responses in general and instruments designed to assess empathy in specific contexts, such as ethnocultural empathy (Rasoal et al., 2011), empathy in the face of anger and pain (Vitaglione and Barnett, 2003; Giummarra et al., 2015), empathy among physicians (Alcorta-Garza et al., 2016), health workers, and patients (Scarpellini et al., 2014) and empathy involved in the relationship between teachers and students (Warren, 2015), among others.

Previous studies, such as systematic reviews, have assessed the psychometric quality of instruments intended to assess empathy in some of these specific contexts. Hemmerdinger et al. (2007) assessed the reliability and validity of scales used to assess empathy in medicine, analyzing 36 different instruments. The Medical Condition Regard Scale, Jefferson Scale of Physician Empathy, Consultation and Relational Empathy, and Four Habits Coding Scheme, which were developed for this specific population, stood out together with Davis Interpersonal Reactivity Index (IRI), Empathy Test, Empathy Construct Rating Scale, and Balanced Emotional Empathy Scale, which assess empathic ability in general, as they presented satisfactory psychometric qualities. However, the authors highlighted that instruments focusing on selecting candidates for the medical program lacked sufficient predictive validity evidence. Nonetheless, they concluded that there were measures with sufficient evidence to investigate the role of empathy in the medical and clinical care fields.

Later, Yu and Kirk (2009) attempted to verify the existence of a gold-standard instrument to assess empathy within the nursing field. They identified 12 instruments, 33.3% of these were originally developed with nursing workers and students (e.g., Empathy Construct Rating Scale and Layton Empathy Test), 33.3% addressed health workers and patients (e.g., Barrett-Lennard Relationship Inventory and Carkhuff Indices of Discrimination and Communication), and 33.3% were developed to assess empathic response in general (e.g., Emotional Empathy Tendency Scale and IRI). The results show that most instruments presented not very robust validity or reliability indicators, while less than 15% of the instruments verified the responsiveness item. The authors concluded that no instrument could be recommended as the gold standard but noted that the Empathy Construct Rating Scale gathered the most robust evidence.

Hong and Han (2020) recently conducted a systematic review to identify scales assessing empathy among health workers in general. Eleven studies were included in the review, among which the Consultation and Relational Empathy, Jefferson Scale of Physician Empathy, and Therapist Empathy Scale (TES). These scales stood out in terms of psychometric quality; however, like previous reviews, the conclusion was that there were no instruments with desirable psychometric qualities to be considered the gold standard. Additionally, none of the measures were specifically developed for professionals working with the elderly, which indicates an important gap in the field.

To our knowledge, no systematic reviews focus on instruments that measure empathic ability in the general population. Hence, this review aimed to describe the psychometric quality and adequacy of instruments available in the literature from 2009 to 2019 to assess empathy in the general population.

## Materials and Methods

**FIGURE 1:**
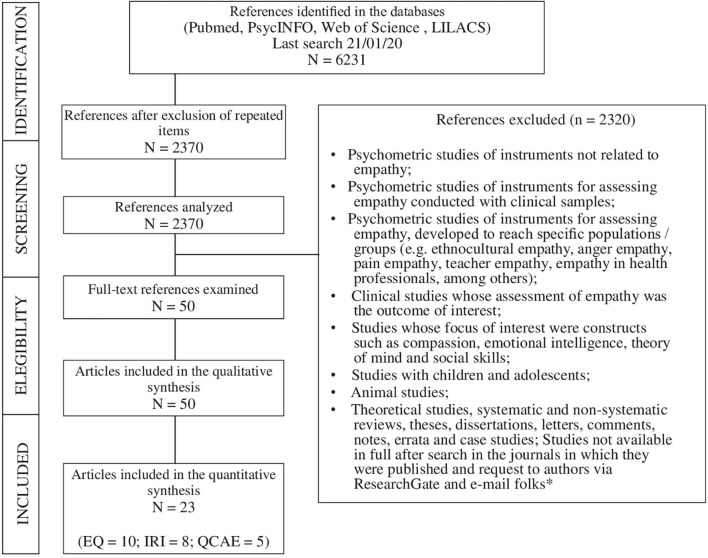
Flowchart describing to the inclusion and exclusion criteria processes based on PRISMA protocol (*Bora and Baysan, 2009; Innamorati et al., 2015).

This study complied with the recommendations proposed by the Preferred Reporting Items for Systematic Review and Meta-Analyses – PRISMA (Moher et al., 2009) and the methodological guidelines established by the BRASIL. Ministério da Saúde et al. (2014). The following databases were searched: PubMed, PsycINFO, Web of Science, Scielo, and LILACS together with the keywords empathy, valid^∗^, reliability, and psychometr^∗^. Inclusion criteria were studies: (a) addressing 18-year-old or older individuals in the general population of both sexes; (b) published between 2009 and 2019, regardless of the language; and (c) with the objective to develop and/or assess the psychometric quality of instruments measuring empathic response in the general population. Figure 1 presents the exclusion criteria and the entire process used to select the studies.

Two mental health workers experienced in psychological and psychometric assessments (FFL, FLO) independently decided on the studies’ eligibility; divergences were resolved by consensus. A standard form was developed to extract the following variables: (a) year of publication; (b) study’s objective; (c) sample characteristics (i.e., country of origin, sample size, sex, age, and education); (d) instrument’s characteristics (objective, number of items, application format, and scoring); and (e) psychometric indicators concerning validity and reliability.

The framework proposed by Andresen (2000) was used to assess the psychometric quality of the papers included in this review. It rates different criteria on a nominal scale ranging from A (strong adequacy) to C (weak or no adequacy), namely: Norms, Standard values; Measurement model; Item/instrument bias; Respondent burden; Administrative burden; Reliability; Validity; Responsiveness; Alternate/accessible forms, and Culture/language adaptations. This review’s authors independently assessed the studies’ psychometric quality and resolved divergences by achieving a consensus. The definitions of psychometric qualities and assessment criteria are presented in Supplementary Material 1.

A qualitative synthesis of the results was performed for each instrument. Additionally, for those instruments with more than two studies, meta-analytic measures of reliability and convergent validity were produced using the Jamovi software. We conduct reliability generalization meta-analysis of Cronbach’s alpha (for the total scale and/or subscales) (Pentapati et al., 2020), and intraclass correlation coefficients (ICC) were grouped for the computation of test-retest meta-analytic measures (Macchiavelli et al., 2020). To group data concerning convergent validity with empathy measures and correlate constructs, we used Pearson/Spearman correlation coefficient (r) as the effect size measure (Duckworth and Kern, 2011). In the case of multiple indicators, the largest indicator in absolute values was chosen. Untransformed estimates and inverse variance weighting were used (Hedges and Olkin, 1985). An average coefficient and a 95% confidence interval (95%CI) were calculated for each meta-analysis. Heterogeneity of the measures between studies was verified using Q-statistic and I2 index. The funnel plot was used to assess the publication bias (Egger et al., 1997).

## Results

Fifty studies were selected, and 23 different instruments were identified. The instruments most frequently addressed were the Empathy Quotient (EQ; *n* = 11), IRI (*n* = 10), and Questionnaire of Cognitive and Affective Empathy (QCAE; *n* = 5). Only one or two studies assessed each of the remaining instruments. A total of 60.9% of the instruments were developed in the period included in this review, from 2009 to 2019. The remaining studies were developed before 2009, and the studies assessed new aspects of their psychometric qualities and/or cross-cultural adaptations. Table 1 presents an overview of each instrument.

**TABLE 1:** Overview of the instruments analyzed to assess general empathic capacity in the general population (ranked from most studied to least studied).

Instrument	Author/year	Objective	Type of application	Type of instrument	No. items	Rating scale	No. of psychometric studies (last 10 years)
Empathy Quotient (EQ-60)	Baron-Cohen and Wheelwright (2004)	To measure the cognitive and affective aspects of empathy	Presential/Online	SRF	60 40 22 15	Likert – 4 points 1 (“strongly disagree”) to 4 (“strongly agree”)	11
Interpersonal Reactivity Index (IRI)	Davis (1983)	To evaluate empathy through 4 factors: fantasy, empathic concern, perspective taking and personal distress.	Presential/Online	SRF	28 26 16	Likert – 5 points 0 (“doesn’t describe me at all”) to 4 (“describes me very well”)	10
Questionnaire of Cognitive and Affective Empathy (QCAE)*	Reniers et al. (2011)	To evaluate empathy through 5 factors: perspective taking, online simulation, emotion contagion, peripherical responsivity and proximal responsivity.	Presential/Online	SRF	31	Likert – 4 points 1 (“strongly disagree”) to 4 (“strongly agree”)	5
The Active-Empathic Listening Scale (AELS)	Drollinger et al. (2006)	Computerized task to evaluate empathy through 3 factors: sensing, processing, responding.	Presential	SRF/HRF	11	Likert – 7 points 1 (“never or almost never true”) to 7 (“always or almost always true”)	2
The Toronto Empathy Questionnaire (TEQ)*	Spreng et al. (2009)	To assess cognitive and affective aspects of empathy	Presential	SRF	16	Likert – 5 points 1 (“never”) to 5 (“always”)	2
Empathy Assessment Index (EAI)*	Gerdes et al. (2011)	To evaluate empathy through 5 factors: affective response, perspective taking, emotion regulation, self-awareness and empathic attitudes.	Online	SRF	54/50	Likert – 5 points 1 (“never”) to 5 (“always”)	2
Affective and Cognitive Measure of Empathy (ACME)*	Vachon and Lynam (2016)	To evaluate empathy through 3 factors: cognitive empathy, affective resonance and affective dissonance	Presential/Online	SRF	36	Likert – 5 points 1 (“strongly disagree”) to 5 (“strongly agree”)	2
The Mexican Empathy Scale (MxES)	Díaz-Loving et al. (1986)	To evaluate empathy through 4 factors: empathic compassion, cognitive empathy, Indifference and disruption	Presential	SRF	32	Likert – 5 points 1 (“does not describe me well”) to (“describes me very well”)	1
Multidimensional Emotional Empathy Scale (MDEES)	Caruso and Mayer (1998)	To evaluate empathy through 6 factors: suffering, positive sharing, responsive crying, emotional attention, feel for other and emotional contagion	Presential	SRF	30	Likert – 5 points 1 (“Strongly disagree”) to 5 (“Strongly agree”)	1
Basic Empathy Scale (BES)	Jolliffe and Farrington (2006)	To assess cognitive and affective aspects of empathy	Presential	SRF	20	Likert – 5 points 1 (“strongly disagree”) to 5 (“strongly agree”)	1
Empathy Inventory (EI)	Falcone et al. (2008)	To evaluate empathy through 4 factors: perspective taking; Interpersonal flexibility; altruism and affective sensitivity	Presential	SRF	40	Likert – 5 points 1 (“never”) to 5 (“always”)	1
Multifaceted Empathy Test (MET)	Dziobek et al. (2008)	Computerized task to measure the cognitive and affective aspects of empathy	Presential	SRF	80	Cognitive test: to select the emotional state of the depicted person out of a set of four possible answers. Affective test: Likert – 9 points 1 (“not at all”) to 9 (“very strongly”)	1
The Vicarious Experience Scale (VES)	López-Pérez et al. (2008)	To evaluate empathy through 2 factors: sympathy and vicarious distress	Presential	SRF	15	Likert – 5 points 1 (“not at all”) to 5 (“extremely”)	1
Interpersonal and Social Empathy Index (ISEI)*	Segal et al. (2013)	To assess cognitive and affective aspects of empathy	Online	SRF	15	Likert – 6 points 1 (“never”) to 6 (“always”)	1
Measure of Empathy and Sympathy (MES)*	Vossen et al. (2015)	To measure empathy and sympathy	Online	SRF	12	Likert – 5 points 1 (“never”) to 5 (“always”)	1
Positive Empathy Scale (PES)*	Morelli et al. (2015)	To evaluate positive empathy	Presential	SRF	7	Likert – 5 points 1 (“does not describe me at all”) to 5 (“describes me very well”)	1
Empathic Behavior Scale (ECE)*	Auné et al. (2017)	To assess the demonstration of the other’s understanding, the ability to put oneself in the other’s place, the ability to support the other, make positive reinforcements through speech, share experiences, take action to reduce conflict and emotional support in painful moments.	Presential	SRF	8	Likert – 5 points 1 (“never”) to 5 (“always”)	1
Empathy Components Questionnaire (ECQ)*	Batchelder et al. (2017)	To evaluate empathy through 5 factors: cognitive ability, cognitive drive, affective ability, affective drive and affective reactivity.	Online	SRF	30	Likert – 4 points 1 (“strongly disagree”) to 4 (“strongly agree”)	1
Empathy Gradient Questionnaire (EGQ)*	Hollar (2017)	To evaluate empathy through 5 factors: family empathy, friend empathy, peer/colleague empathy, distant other empathy, and species empathy.	Presential	SRF	23	Likert – 5 points 1 (“I very much disagree”) to 5 (“I very much agree”)	1
Cognitive, Affective, and Somatic Empathy Scales (CASES)*	Raine et al. (2018)	To evaluate empathy through 3 factors: cognitive, affective, and somatic empathy	Presential	SRF	30	Likert – 4 points 0 (“rarely”) to 3 (“often”)	1
Online Empathy Questionnaire (QoE)*	Miguel et al. (2018)	To evaluate empathy through 3 factors: responsiveness, respect and availability.	Online	SRF	23	Likert – 5 points 1 (“never”) to 5 (“always”)	1
Pictorial Empathy Test (PET)*	Lindeman et al. (2018)	Computerized task to evaluate affective empathic	Online	SRF	7	Likert – 5 points 1 (“not at all”) to 5 (“always”)	1
Single Item Trait Empathy Scale (SITES)*	Konrath et al. (2018)	To measure empathy through a single item	Online	SRF	1	Likert – 5 points 1 (“not very true of me”) to 5 (“very true of me”)	1

SRF, self-report form; HRF, hetero-report form; *new instruments proposed from 2009.

Table 1 shows that most instruments are self-report scales (*n* = 21), rated on a Likert scale (70% included five-point scales), with the number of items ranging from one to 80 (median = 23). Three instruments present alternative versions with fewer items [EQ, IRI, and Empathy Assessment Index (EAI)]. In most cases, data were collected face-to-face (*n* = 12), while the Active-Empathic Listening Scale (AELS) (Drollinger et al., 2006) was the only instrument with an other-report version. Two instruments consisted of computational tasks with the presentation of photorealistic stimuli: the Multifaceted Empathy Test (MET) (Dziobek et al., 2008) and the Pictorial Empathy Test (PET) (Lindeman et al., 2018). Data concerning the samples used by the different studies are presented in Table 2.

**TABLE 2:** Characterization of the samples used by the different studies (*N* = 50 – ranked from most studied to least studied).

Instrument	Author/year	Country	Sample		
			N/Sex	Age-years (x̄/DP)	Level of education
Empathy Quotient (EQ) (11 studies)	Kim and Lee (2010)	Canada	322♀/156♂	27,2 (NI)	44% CS/56% UD
	Preti et al. (2011)	Italy	138♀/118♂	24.0 (4.5)	43% HS/30.5% ES/26.5% UD
	Rodrigues et al. (2011)	Portugal	329♀/177♂	33.9 (11.7)	Not informed
	Gouveia et al. (2012)	Brazil	134♀/103♂	31.0 (14.1)	52.3% CS
	Wright and Skagerberg (2012)	United States	2126♀/3060♂	45 to 54	Not informed
	Kosonogov (2014)	Russia	121♀/100♂	24.9 (7.7)	33% CS/33% UD
	Senese et al. (2016)	Italy	409♀/224♂	24.3 (5.9)	Not informed
	Kose et al. (2018)	Turkey	195♀/241♂	22.6 (7.2)	89.9% CS
	Redondo and Herrero-Fernández (2018)	Spain	350♀/121♂	20.0 (2.0)	CS
	Zhang et al. (2018)	China	754♀/884♂	EFA group: 20.7 (2.1)/CFA group: 21.7 (3.2)	CS
	Zhao et al. (2018)	China	375♀/213♂	24.1 (6.2)	15.4 (2.2 years)
Interpersonal Reactivity Index (IRI) (10 studies)	Fernández et al. (2011)	Chile	234♀/201♂	20.1 (1.9)	CS
	Sampaio et al. (2011)	Brazil	S1: 176♀/74♂/S2: 144♀/107♂	S1: 20.8 (1.9)/S2 = 20.8 (1.9)	CS
	Gilet et al. (2013)	Switzerland	190♀/132♂	49.5 (21.1)	UN
	Koller and Lamm (2014)	Austria	1203♀♂	18 to 81	54.6% CS
	Braun et al. (2015)	Belgium	S1: 710♀/534♂/S2: 425♀/304♂	S1: 19.6 (1.6)/S2: 19.3 (1.5)	S1: CS/S2: CS
	Formiga et al. (2015)	Brazil	254♀/397♂	21.1 (2.2)	CS
	Chrysikou and Thompson (2016)	United States	246♀/171♂	33.2 (NI)	Not informed
	Ingoglia et al. (2016)	Italy	S1: 773♀/331♂/S2: 435♀/401♂/S3: 389♀/260♂	S1:17.6 (3.0)/S2:20.5 (3.3)/S3: 20.5 (3.3)	S1: 74.1% HS/S2: 42.3% HS/S3: 42.1% HS
	Budagovskaia et al. (2017)	Russia	S1: 43♀/160♂/S2: 217♀/101♂	S1: 17 to 25/S2: NI	S1: CS/S2: NI
	Lucas-Molina et al. (2017)	Spain	S1: 1780♀/719 ♂S2: 607♀/831♂	S1: 21.1 (3.6)/S2: 40.0 (5.4)	S1: CS/S2: NI
Questionnaire of Cognitive and Affective Empathy (QCAE) (5 studies)	Reniers et al. (2011)	United Kingdom	S1 = 434♀/206♂/S2 = 230♀/88♂	S1 = 23.7 (7.8)/S2 = 30.0 (11.0)	45% UD
	Di Girolamo et al. (2017)	Italy	S1: 300♀/107♂; QS2: 224♀/61♂	S1: 22.6 (4.6)/S2: 26.4 (7.0)	S1: CS/S2: 60% CS
	Myszkowski et al. (2017)	France	275♀/143♂	26.1 (8.2)	77.5% CS
	Queirós et al. (2018)	Portugal	413♀/149♂	27.5 (10.3)	Not informed
	Liang et al. (2019)	China	615♀/609♂	22.16 (2.93)	CS
The Active-Empathic Listening Scale (AELS) (2 studies)	Bodie (2011)	United States	S1 = 250♀/165♂/S2 = 111♀/106♂	S1 = 20.0 (3.0)/S2 = 20.5 (2.2)	S1: 98.3% HS/S2: 98.1% HS
	Gearhart and Bodie (2011)	United States	191♀/154♂	20,3 (2.9)	CS
The Toronto Empathy Questionnaire (TEQ) (2 studies)	Spreng et al. (2009)	Canada	S1 = 100♀/10/♂S2 = 55♀/ 24♂/S3 = 46♀/19♂	S1: 18.8 (1.2)/S2: 18.9 (3.0)/S3: 18.6 (2.3)	S1: CS/S2: CS/S3: CS
	Totan et al. (2012)	Turkey	357♀/231♂	20.6 (NI)	UN
Empathy Assessment Index (EAI) (2 studies)	Gerdes et al. (2011)	United States	260♀/50♂	18 to 60 (NI)	CS
	Lietz et al. (2011)	United States	587♀/186♂	21,4 (NI)	88.1% CS
Affective and Cognitive Measure of Empathy (ACME) (2 studies)	Vachon and Lynam (2016)	United States	S1: 162♀/207♂/S2: 340♀/368♂/S3: 120♀/90♂	S1: NI/S2: NI/S3: NI	S1: CS/S2: CS/S3: CS
	Murphy et al. (2018)	United States	215♀/186♂	35.5 (11.0)	Not informed
The Mexican Empathy Scale (MxES) (1 study)	Mendez et al. (2011)	United States	S1 = 344♀/131♂/S2 = 102♀/ 34♂/S3 = 50♀/29♂ S4 = 104♀/59♂	S1 = NI/S2 = 18 to 45/S3 = 20.3 (NI)/S4 = 18 to 22	CS
Multidimensional Emotional Empathy Scale (MDEES) (1 study)	Alloway et al. (2016)	United States	197♀/115♂	19.0 (NI)	CS
Basic Empathy Scale (BES) (1 study)	Carré et al. (2013)	France	260♀/110♂	26.05 (12.4)	67% CS
Empathy Inventory (EI) (1 study)	Falcone et al. (2013)	Brazil	129♀/101♂	26.2 (11.8)	50.4% CS
Multifaceted Empathy Test (MET) (1 study)	Foell et al. (2018)	United States	58♀/22♂	19.6 (NI)	CS
The Vicarious Experience Scale (VES) (1 study)	Oceja Fernández et al. (2009)	Spain	S1 = 220♀/160♂/S2 = 44♀/ S3 = 19♀/21♂	S1 = 32.7 (13.8)/S2 = NI/S3 = 33.6 (13.0)	S1: UD/S2 = CS/S3 = 67% UD
Interpersonal and Social Empathy Index (ISEI) (1 study)	Segal et al. (2013)	United States	296♀/152♂	23.0 (5.7)	UN
Measure of Empathy and Sympathy (MES) (1 study)	Wang et al. (2017)	China	388♀/220♂	20.6 (2.6)	78.3% CS/21.7% UD
Positive Empathy Scale (PES) (1 study)	Yue et al. (2016)	China	S1: 428♀♂/S2: 503♀♂	Not informed	CS
Empathic Behavior Scale (ECE) (1 study)	Auné et al. (2017)	Argentina	946♀/222♂	22.0 (6.0)	CS
Empathy Components Questionnaire (ECQ) (1 study)	Batchelder et al. (2017)	United States	S1: 66♀/35♂. S2: 116♀/95♂	S1: 20.3♀ (1.9)/20.40♂ (1.9)/S2: 29.2♀ (9.9)/26.0♂ (6.6)	S1: CS/S2: 47.4% CS
Empathy Gradient Questionnaire (EGQ) (1 study)	Hollar (2017)	United States	78♀/83♂	18 to 60	29.8% CS/70.2% UD
Cognitive, Affective, and Somatic Empathy Scales (CASES) (1 study)	Park et al. (2019)	China	172♀/176♂	22.7♀ (NI);/21.5♂ (NI)	CS
Online Empathy Questionnaire (QoE) (1 study)	Miguel et al. (2018)	Brazil	2727♀/2074♂	27.73 (7.9)	37.5% HS or less/49.2% CS/13.3% UD
Pictorial Empathy Test (PET) (1 study)	Lindeman et al. (2018)	Finland	S1: 49♀/42♂/S2: 2035♀/1049♂ S3: 65♀/49♂	S1: 49.9 (NI)/S2: 27.6 (8.8)/S3: 31.0 (11.2)	S1: Not informed/S2: 54.2% CS/S3: 48.2% CS
Single Item Trait Empathy Scale (SITES) (1 study)	Konrath et al. (2018)	United States	5724♀♂	36.0 (12.1)	Not informed

*CS, college students; ES, elementary school; HS, high school; NS, non-student; UD, university degree ♀, women; ♂, men; S, sample.*

As shown in Table 2, the smallest sample was composed of 50 participants, and the largest sample had 5.724 participants (mean = 1036.6 ± 1577.5). Regarding age, most studies addressed young/middle-aged adults (median = 24.0); with varied educational levels (college students and individuals with a university degree = 67.3%). As for the countries of origin, European countries predominated (*n* = 20), followed by North American countries (*n* = 17), Asian (*n* = 6), and South American countries (*n* = 7).

The instruments’ psychometric proprieties were assessed according to the parameters proposed by Andresen (2000). The results of which are presented in Tables 3, 4 and Supplementary Material 2 present raw data concerning these indicators based on reliability and validity criteria (construct and criterion).

**TABLE 3:** Analysis of psychometric qualities by the different instruments, according to the studies analyzed (*n* = 50) – Part A.

Instrument	Study	Norms/standard values	Measurement model	Item/Instrument Bias	Burden (1/2)	Reliability	Alternative/accessible forms	Culture/Language adaptation
Empathy Quotient (EQ)	Kim and Lee (2010) EQ-40	B	A	Face validity: B	A/A	Internal consistency: B Test-retest: A	C	B (Korean)
	Preti et al. (2011) EQ-60	B	Unvalued	Not applicable	A/A	Internal consistency: B Test-retest:A	C	B (Italian)
	Rodrigues et al. (2011) EQ-22	Unvalued	Unvalued	Face validity: A	A/A	Internal consistency: A	C	B (Portuguese)
	Gouveia et al. (2012) EQ-15	Unvalued	Unvalued	Face validity: A	A/A	Internal consistency: C Composite reliability: B	C	B (Brazilian version)
	Wright and Skagerberg (2012) EQ-40	B	Unvalued	Change version: A Unchanged version:B	A/A	Internal consistency changed version: A unchanged version: A	B computerized	Not applicable
	Kosonogov (2014) EQ-60	B	A	Face validity: C	A/A	Internal consistency – EQ-60 – 1 Factor: A EQ-29 – 3 factors: A EQ-28 – Lawrence et al. model – 3 factors: B EQ-21 – 3 factors: B EQ-15 – 3 factors: B EQ-15 – Muncer and Ling model – 3 factors: C Test-retest: EQ-60: A	C	B (Russian version)
	Senese et al. (2016) EQ-40	B	Unvalued	Not applicable	A/A	Internal consistency: B	B computerized	B (Italian version)
	Kose et al. (2018) EQ-60	B	Unvalued	Face validity: B	A/A	Internal consistency: B Test-retest: A	C	B (Turkish version)
	Redondo and Herrero-Fernández (2018) EQ-60	Unvalued	Unvalued	Not applicable	A/A	Unvalued	C	B (Spanish version)
	Zhang et al. (2018) EQ-60	B	Unvalued	Face validity: A	A/A	Internal consistency: B Test-retest: A	C	B (Chinese version)
	Zhao et al. (2018) EQ-60	B	Unvalued	Face validity: A	A/A	Internal consistency: EQ-40: A/EQ-15: A Test-retest: EQ-40: A/EQ-15:B	B computerized	B (Chinese version)
Interpersonal Reactivity Index (IRI)	Fernández et al. (2011) IRI-28	B	Unvalued	Face validity: B	A/A	Internal consistency: B Test-retest: A	C	B (Spanish version)
	Sampaio et al. (2011) IRI-28	B	Unvalued	Face validity: B	A/A	Internal consistency: B	C	B (Brazilian version)
	Gilet et al. (2013) IRI-28	B	Unvalued	Face validity: A	A/A	Internal consistency: B Test-retest: A	C	B (French version)
	Koller and Lamm (2014) IRI-28	Unvalued	A	B	A/A	Internal consistency: B	C	B (German)
	Braun et al. (2015) IRI-28	B	Unvalued	Face validity: B	A/A	Internal consistency: B	C	B (French version)
	Formiga et al. (2015) IRI-26	Unvalued	Unvalued	Not applicable	A/A	Internal consistency:B	C	B (Brazilian version)
	Chrysikou and Thompson (2016) IRI-28	B	Unvalued	Not applicable	A/A	Unvalued	B computerized	Not applicable
	Ingoglia et al. (2016) IRI-28/IRI-16	B	A	Not applicable	A/A	Internal consistency – IRI-28/IRI-16:B	C	Not applicable
	Budagovskaia et al. (2017) IRI-28	B	Unvalued	Face validity: A	A/A	Internal consistency: B	C	B (Russian version)
	Lucas-Molina et al. (2017) IRI-28	Unvalued	Unvalued	Not applicable	A/A	Internal consistency: B	C	B (Spanish)
Questionnaire of Cognitive and Affective Empathy (QCAE)	Reniers et al. (2011)	Unvalued	Unvalued	Face validity: B	A/A	Internal consistency: B	C	Not applicable
	Di Girolamo et al. (2017)	B	Unvalued	Not applicable	A/A	Internal consistency: Paper-pencil and Online total: A/subscales: B	A paper-pencil	Not applicable
	Myszkowski et al. (2017)	Unvalued	Unvalued	Face validity: B	A/A	Internal consistency: B	C	B (French version)
	Queirós et al. (2018)	B	Unvalued	Face validity: B	A/A	Internal consistency total: A/subscales: B	C	B (Portuguese version)
	Liang et al. (2019)	B	Unvalued	Face validity: A	A/A	Internal consistency total: A/subscales: B Test-retest total: A/subscales: B	C	B (Chinese version)
The Active-Empathic Listening Scale (AELS)	Bodie (2011)	Unvalued	Unvalued	Face validity: C	A/A	Internal consistency total: A/subscales: B	B other-report	Not applicable
	Gearhart and Bodie (2011)	Unvalued	Unvalued	Not applicable	A/A	Not applicable	C	Not applicable
The Toronto Empathy Questionnaire (TEQ)	Spreng et al. (2009)	B	Unvalued	Face validity: C	A/A	Internal consistency: A	C	Not applicable
	Totan et al. (2012)	Unvalued	Unvalued	Face validity: B	A/A	Internal consistency: B Test-retest: B	C	B (Turkish version)
Affective and Cognitive Measure of Empathy (ACME)	Vachon and Lynam (2016)	B	Unvalued	Face validity: C	A/A	Internal consistency: A	C	Not applicable
	Murphy et al. (2018)	Unvalued	Unvalued	Not applicable	A/A	Not applicable	C	Not applicable
Empathy Assessment Index (EAI)	Gerdes et al. (2011)	Unvalued	Unvalued	Face validity:A	A/A	Internal consistency: A Test-retest: B	C	Not applicable
	Lietz et al. (2011)	Unvalued	Unvalued	Not applicable	A/A	Internal consistency: B Test-retest: B	C	Not applicable
Measure of Empathy and Sympathy (MES)	Wang et al. (2017)	Unvalued	Unvalued	Not applicable	A/A	Internal consistency total: B/subscales: B	C	B (Adult version)
Multidimensional Emotional Empathy Scale (MDEES)	Alloway et al. (2016)	B	Unvalued	Not applicable	A/A	Internal consistency: A	C	Not applicable
Basic Empathy Scale (BES)	Carré et al. (2013)	B	Unvalued	Not applicable	A/A	Internal consistency: 2 Factors:B/3 Factors: B Test-retest: 2 factors: B/3 factors: B	C	B (Adult version)
Empathy Inventory (EI)	Falcone et al. (2013)	Unvalued	Unvalued	Not applicable	A/A	Not applicable	C	Not applicable
Multifaceted Empathy Test (MET)	Foell et al. (2018)	Unvalued	Unvalued	Face validity: A	B/B	Internal consistency Cognitive empathy (before/after reduction): C Emotional empathy: A	C	B (English version)
The Vicarious Experience Scale (VES)	Oceja Fernández et al. (2009)	Unvalued	Unvalued	Face validity: C	A/A	Internal consistency: B	C	Not applicable
Interpersonal and Social Empathy Index (ISEI)	Segal et al. (2013)	Unvalued	Unvalued	Face validity: A	A/A	Internal consistency total:A/subscales: B	C	Not applicable
The Mexican Empathy Scale (MxES)	Mendez et al. (2011)	B	Unvalued	Face validity: A	A/A	Internal consistency: B	C	B (English version)
Positive Empathy Scale (PES)	Yue et al. (2016)	Unvalued	Unvalued	Face validity: A	A/A	Internal consistency: A Test-retest:A	C	B (Chinese version)
Empathic Behavior scale (ECE)	Auné et al. (2017)	Unvalued	Unvalued	Face validity: C	A/A	Internal consistency: A	C	Not applicable
Empathy Components Questionnaire (ECQ)	Batchelder et al. (2017)	B	Unvalued	Face validity: A	A/A	Internal consistency: B	C	Not applicable
Empathy Gradient Questionnaire (EGQ)	Hollar (2017)	B	Unvalued	Face validity: C	A/A	Internal consistency: A	C	Not applicable
Cognitive, Affective, and Somatic Empathy Scales (CASES)	Park et al. (2019)	Unvalued	Unvalued	Face validity: B	A/A	Internal consistency 1 and 2 Factors: A 3 factors: B	C	B (Chinese version for adults)
Online Empathy Questionnaire (QoE)	Miguel et al. (2018)	Unvalued	Unvalued	Face validity: C	A/A	Internal consistency total: A/subscales: B Test-retest: A	C	Not applicable
Pictorial Empathy Test (PET)	Lindeman et al. (2018)	Unvalued	Unvalued	Face validity: C	A/A	Internal consistency: A	C	Not applicable
Single Item Trait Empathy Scale (SITES)	Konrath et al. (2018)	B	Unvalued	Face validity: C	A/A	Test-retest: B	C	Not applicable

*1-Respondent; 2-Administrative.*

**TABLE 4:** Analysis of psychometric qualities by the different instruments according to the studies analyzed (*N* = 50) – Part B.

Instrument	Study	Factor Structure	Convergent/Discriminant	Known groups	Predictive/Responsiveness
Empathy Quotient (EQ)	Kim and Lee (2010) EQ-40	CFA – EQ-40: 3 Factors Model: C CFA – EQ-28: 1 Factor Model: C CFA – EQ-28: Lawrence et al. – 3 Factors Model: B CFA – EQ-15: Muncer and Ling – 3 Factors Model: A	Convergent: B	Gender: B	Not evaluated
	Preti et al. (2011) EQ-60	CFA – EQ-60: 3 Factors Model: B	Convergent (related construct): B Discriminant: A	Gender: A	Not evaluated
	Rodrigues et al. (2011) EQ-22	EFA – EQ-22: 4 Factors Model: B	Discriminant: A	Not evaluated	Not evaluated
	Gouveia et al. (2012) EQ-15	CFA – EQ-15: 1 Factor Model: C CFA – EQ-15 – Muncer and Ling – 3 Factors Model: C	Not evaluated	Age: A Gender: A Education: C	Not evaluated
	Wright and Skagerberg (2012) EQ-40	Not evaluated	Convergent: B Convergent (related construct): B Discriminant: A	Gender: A	Not evaluated
	Kosonogov (2014) EQ-60	EFA: B CFA – EQ-60: 1 Factor Model: C CFA – EQ-29 – 3 Factors Model: C CFA – EQ-28 – Lawrence et al. – 3 Factors Model: C CFA – EQ-21 – 3 Factors Model: C CFA – EQ-15 – 3 Factors Model: B CFA – EQ-15 – Muncer and Ling – 3 Factors Model: B CFA – EQ-14 – 1 Factor Model: A	Convergent: B	Gender: A	Not evaluated
	Senese et al. (2016) EQ-40	CFA – EQ-40 – 1 Factor Model: C CFA – EQ-28 – 1 Factor Model: A CFA – EQ-28 – Lawrence et al. – 3 Factors Model: A CFA – EQ-15 – 1 Factor Model: C CFA – EQ-15 – Muncer and Ling – 3 Factors Model: A Measurement invariance: gender	Convergent: B Convergent (related construct): B Discriminant: A	Gender: B	Not evaluated
	Kose et al. (2018) EQ-60	EFA: B	Convergent: C	Gender: A	Not evaluated
	Redondo and Herrero-Fernández (2018) EQ-60	CFA – EQ-28 – Lawrence et al. – 3 Factors Model: A CFA – EQ-23 – 3 Factors Model (factor load > 0.20): A CFA – EQ-23 – 1 Factor Model (factor load > 0.20): A CFA – EQ-15 – Kim and Lee – 3 Factors Model: C CFA – EQ-15 – 1 Factor Model: A	Convergent: B Convergent (related construct): B	Gender: B	Not evaluated
	Zhang et al. (2018) EQ-60	CFA – EQ-29 – 4 Factors Model: A	Not evaluated	Gender: A	Not evaluated
	Zhao et al. (2018) EQ-60	CFA – EQ-40 – 1 Factor Model: C CFA – EQ-40 – Lawrence et al. – 3 Factors Model: C CFA – EQ-22 – Wakabayashi et al. – 1 Factor Model: C CFA – EQ-15 – Muncer and Ling – 3 Factors Model: C CFA – EQ-26 – Alisson et al. – 2 Factors Model: A CFA – EQ-15 – Modified Guan et al. – 1 Factor Model: A CFA – EQ-15 – Guan et al. – 1 Factor Model: A	Convergent: B Convergent (related construct): B	EQ-40: gender: A EQ-15: gender: C	Not evaluated
Interpersonal Reactivity Index (IRI)	Fernández et al. (2011) IRI-28	CFA – Davis Original 4 Factors Model: C CFA – Cliffordson 2° order 4 Factors Model: C	Convergent (related construct): B	Gender: A	Not evaluated
	Sampaio et al. (2011) IRI-28	EFA – 26 items – 4 Factors Model: B CFA – 1 Factor Model: A CFA – 2 Factors Model: A CFA – Davis Original 4 Factors Model: A	Not evaluated	Gender: A	Not evaluated
	Gilet et al. (2013) IRI-28	CFA – 1 Factor Model: C CFA – 2 Factors Model: C CFA – Davis Original 4 Factors Model: C	Convergent: B	Gender: A age: B	Not evaluated
	Koller and Lamm (2014) IRI-28	CFA – 3 Factors Model: A CFA – Davis Original 4 Factors Model: A CFA – 5 Factors Model: A Measurement invariance: gender and age	Not evaluated	Not evaluated	Not evaluated
	Braun et al. (2015) IRI-28	Sample 1: EFA – IRI-15 – 4 Factors Model: B CFA – IRI-15 – 4 Factors Model: A CFA – Davis Original Model – 4 Factors: B Sample 2: CFA – IRI-15 – 4 Factors Model: A Measurement invariance: gender	Convergent (related construct): B	Gender: A	Not evaluated
	Formiga et al. (2015) IRI-26	EFA – Ribeiro et al. – 3 Factors Model: A EFA – Sampaio et al. 4 Factors Model: A	Not evaluated	Not evaluated	Not evaluated
	Chrysikou and Thompson (2016) IRI-28	CFA – 2 Factors Model: C CFA – Pulos et al. – 3 Factors Model: B CFA – Davis Original 4 | Factors Model: B	Not evaluated	Gender: A	Not evaluated
	Ingoglia et al. (2016) IRI-28 IRI-16	Sample 1: EFA – 16 items – 4 Factors Model: B Sample 2: CFA – 16 items – 4 Factors Model: A Sample 3: CFA – 16 items – 4 factors: A Measurement invariance: gender and age	Convergent (related construct): A	Gender: A	Not evaluated
	Budagovskaia et al. (2017) IRI-28	EFA – Davis Original 4| Factors Model: C	Convergent: A Convergent (related construct): B	Gender: A	Not evaluated
	Lucas-Molina et al. (2017) IRI-28	CFA – Davis Original 4 Factors Model: C CFA – Hawk et al: 4 Factors 2nd order Model: C Measurement invariance: gender	Not evaluated	Gender: B	Not evaluated
Questionnaire of Cognitive and Affective Empathy (QCAE)	Reniers et al. (2011)	EFA: B CFA – 5 Factors Model – 1st order: A CFA – 5 Factors Model – 2nd order: A	Convergent: A Convergent (related construct): B	Gender: A	Not evaluated
	Di Girolamo et al. (2017)	CFA – Paper-pencil scales dataset: 1 Factor Model: C Reniers et al. Original 5 Factors Model – 1st order: A Reniers et al. Original 5 Factors Model – 2nd order: A CFA – Online dataset 1 Factor Model: C Reniers et al. Original 5 Factors Model – 1st order: A Reniers et al. Original 5 Factors Model – 2nd order: A Measurement invariance: paper-pencil x online version	Convergent (related construct): Paper-pencil scales dataset: B Online dataset: B	Not evaluated	Not evaluated
	Myszkowski et al. (2017)	CFA – Reniers et al. Original 5 Factors Model: A CFA – 5 Factors Model – 2nd order: A CFA – 5 Factors Model (1 inequality constraint): A CFA – 5 Orthogonal Factors Model: C CFA – 5 Factors Model– 2nd order (1 inequality constraint): C CFA – 1 Factor Model: C CFA – 2 Orthogonal Factors Model: C CFA – 2 Correlated Factors Model: C Measurement invariance: gender	Not evaluated	Not evaluated	Not evaluated
	Queirós et al. (2018)	CFA – Reniers et al. Original 5 Factors Model: B CFA – 5 Factors Model – with 2 correlated 2nd order: B Measurement invariance: gender	Not evaluated	Gender: A	Not evaluated
	Liang et al. (2019)	EFA: B CFA – Reniers et al. Original 5 Factors Model: A CFA – 5 Factors Model – 2nd order: A CFA – 4 Factors Model – 1st order: A CFA – 4 Factors Model – 2nd order: A Measurement invariance: gender	Convergent: A Convergent (related construct): C	Gender: A	Not evaluated
The Active-Empathic Listening Scale (AELS)	Bodie (2011)	Sample 1: CFA – self-report – 3 Factors Model: A Sample 2: CFA – other-report – 3 Factors Model: B FIA – Self-report x Other-report – 3 Factors Model: A Measurement invariance: self and other-report version (1st-order factor: measurement weights; 2nd-order factor: structural weights)	Self-report: Convergent: B Convergent (related construct): A Other-report: Convergent (related construct): A	Good/bad listener:A	Not evaluated
	Gearhart and Bodie (2011)	CFA – 3 Factors Model: A	Convergent (related construct): B	Not evaluated	Not evaluated
The Toronto Empathy Questionnaire (TEQ)	Spreng et al. (2009)	Sample 1/2: EFA: B	Convergent: A Convergent (related construct): B	Gender: A	Not evaluated
	Totan et al. (2012)	EFA – TEQ-16: 1 Factor Model: B EFA – TEQ-13: 1 Factor Model: C CFA – TEQ-13: 1 Factor Model: A CFA – TEQ-13: 1 Factor Model (with error covariances):A CFA – TEQ-16: 1 Factor Model: A	Convergent: A	Gender: A	Not evaluated
Affective and Cognitive Measure of Empathy (ACME)	Vachon and Lynam (2016)	Sample1/2: CFA – 3 Factors Model: A Measurement invariance: gender	Convergent: A Convergent (related construct): B	Not evaluated	Not evaluated
	Murphy et al. (2018)	CFA – 3 Factors Model: C CFA – 5 Factors Model: A ESEM – 3 Factors Model: A ESEM – 4 Factors Model: A	Convergent: A Convergent (related construct): A	Not evaluated	Not evaluated
Empathy Assessment Index (EAI)	Gerdes et al. (2011)	EFA: B	Convergent: A	Not evaluated	Not evaluated
	Lietz et al. (2011)	1st half of the sample CFA – EAI-40 – 5 Factors Model: C CFA – EAI-24 – 5 Factors Model: B CFA – EAI-24 – 5 Factors Model (with eight error covariance): A CFA – EAI-17 – 5 Factors Model: A CFA – EAI-17 – 5 Factors Model – with correlated error: A 2nd half of the sample CFA – EAI-24 – 5 Factors Model: C CFA – EAI-17 – 5 Factors Model: B CFA – EAI-17 – 5 Factors Model (with eight error covariance): A	Convergent (related construct): B	Gender: B Race: B College majors: B Socioeconomic: B	Not evaluated
Measure of Empathy and Sympathy (MES)	Wang et al. (2017)	EFA: A CFA – 3 Factors Model: A	Convergent: B Convergent (related construct): B	Gender: A	B
Multidimensional Emotional Empathy Scale (MDEES)	Alloway et al. (2016)	EFA: B	Convergent (related construct):C	Not evaluated	Not evaluated
Basic Empathy Scale (BES)	Carré et al. (2013)	CFA – BES – 1 Factor Model – 20 items: B CFA – BES – 2 Factors Model – 20 items: A CFA – BES – 3 Factors Model – 20 items: A CFA – BES – 3 Factors Model – 19 items: A	Convergent: A Convergent (related construct):C	Gender: A	Not evaluated
Empathy Inventory (EI)	Falcone et al. (2013)	Not evaluated	Convergent: A	Not evaluated	Not evaluated
Multifaceted Empathy Test (MET)	Foell et al. (2018)	Not evaluated	Convergent: B Convergent (related construct): B	Not evaluated	Not evaluated
The Vicarious Experience Scale (VES)	Oceja Fernández et al. (2009)	EFA: C	Convergent: B Discriminant: A	Not evaluated	C
Interpersonal and Social Empathy Index (ISEI)	Segal et al. (2013)	EFA – 1 Factor Model: C EFA – Uncorrelated 4 Factors Model: C EFA – Correlated 4 Factors Model: B	Not evaluated	Not evaluated	Not evaluated
The Mexican Empathy Scale (MxES)	Mendez et al. (2011)	EFA: B	Convergent: A	Not evaluated	C
Positive Empathy Scale (PES)	Yue et al. (2016)	EFA: 1 Factor Model: A CFA: 1 Factor Model: B	Convergent: B Convergent (related construct): B discriminant: A	Not evaluated	Not evaluated
Empathic Behavior Scale (ECE)	Auné et al. (2017)	EFA: A	Convergent (related construct): B	Gender: A	Not evaluated
Empathy Components Questionnaire (ECQ)	Batchelder et al. (2017)	Sample 1: EFA: B Sample 2: CFA – Model 1: C CFA – Model 2: C CFA – Model 3: C CFA – Model 4: B	Not evaluated	Gender: A	B
Empathy Gradient Questionnaire (EGQ)	Hollar (2017)	CFA – 1 Factor Model: B CFA – 5 Factors Model: A	Not evaluated	Age: C Gender: C Race: C	Not evaluated
Cognitive, Affective, and Somatic Empathy Scales (CASES)	Park et al. (2019)	CFA – 1 Factor Model: B CFA – 2 Factors Model: B CFA – 3 Factors Model: B	Convergent: A Convergent (related construct): A	Not evaluated	Not evaluated
Online Empathy Questionnaire (QoE)	Miguel et al. (2018)	Not evaluated	Convergent (related construct): A	Gender: A	Not evaluated
Pictorial Empathy Test (PET)	Lindeman et al. (2018)	CFA – 1 Factor Model: B CFA – 1 Factor Model (with correlated error): A	Convergent: B Convergent (related construct): B discriminant: A	Gender: A	Not evaluated
Single Item Trait Empathy Scale (SITES)	Konrath et al. (2018)	Not evaluated	Convergent: B Convergent (related construct): B	Gender: A	Not evaluated

*CFA, confirmatory factor analysis; EFA, exploratory factor analysis; ESEM, exploratory structural equation model.*

### Empathy Quotient

Eleven studies (22.0%) assessed the EQ’s psychometric properties, six of which applied the instrument’s complete version (60 items); three applied the 40-item version (filler items are removed); one study applied the 22-item version, and one the 15-item version.

The Respondent burden criterion was considered satisfactory in all the studies and received grade A; all the versions were brief and well accepted by the target population. Administrative burden also received grade A because the EQ is easy to apply, score, and interpret. None of the studies presented specific normative indicators such as the T score or percentile distribution, only data concerning the mean score (*n* = 8), which resulted in grade B.

Regarding the Measurement model criterion, only Kim and Lee (2010; EQ-40) and Kosonogov (2014; EQ-60) presented kurtosis and asymmetry indicators to show the normality of data distribution. The remaining studies (81.8%) did not report analyses with this purpose, revealing a weakness regarding this psychometric indicator.

Seven studies conducted the EQ cross-cultural adaptation into Korean, Portuguese (Portugal and Brazil), Russian, Turkish, and Chinese. Rodrigues et al. (2011), Gouveia et al. (2012), Zhang et al. (2018), and Zhao et al. (2018) obtained grade A in the Item/instrument bias criterion as they adopted the recommended guidelines for face validity, namely: translation, back translation, peer review, and pretest applied in the target population (Beaton et al., 2000). The Korean and Turkish versions (Kim and Lee, 2010; Kose et al., 2018) received grade B because these did not report a pretest. The Russian version (Kosonogov, 2014) obtained grade C because it did not report its procedures. Two other studies (Preti et al., 2011 and Senese et al., 2016) assessed the psychometric quality of the Italian version, using the version previously adapted by Baron-Cohen (2004), while Redondo and Herrero-Fernández (2018) analyzed the properties of the version previously adapted by Allison et al. (2011) into Spanish.

The internal consistency of the 60-item version presented alpha values that ranged from 0.76 to 0.85; most obtained grade B (*N* = 3). Even with a smaller number of items, the short versions maintained alpha values within a similar pattern (0.78 to 0.87). The cumulative alpha for total scale was 0.85 (IC95%: 0.81 – 0.85) with moderate heterogeneity (I2 = 47.96%; *Q* = 23.866, *p* = 0.02). The funnel plot showed asymmetry (Egger’s: *p* < 0.001) (see Supplementary Material 3.1). Subgroup analyzes considering the instrument’s different version indicate cumulative alpha values of 0.76 (CI95%: 0.74–0.79; I2 = 7.52%; *Q* = 2.166, *p* = 0.34) for the 60-item version, 0.84 (CI95%: 0.78–0.89; I2 = 97.3%; *Q* = 37.044, *p* < 0.001) for the 40-item version, and 0.81 (CI95%: 0.74–0.87; I2 = 96.4%; *Q* = 49.668, *p* < 0.001) for the 15-item version.

The studies concerning temporal stability used the test-retest methodology (*N* = 6), with intervals between 1 and 4 weeks, and indicated excellent indexes (estimated average correlation coefficient: 0.89 (CI95%: 0.83–0.94); I2 = 90.76%; *Q* = 22.330, *p* = 0.001; Egger’s: *p* < 0.001) (see Supplementary Material 3.2).

Regarding convergent validity, the studies used the IRI, Self-assessed Empathizing, Questionnaire Measure of Emotional Empathy, and Quotient of Empathic Abilities as a reference and found weak to moderate correlations (most obtained grade B, with correlations between 0.30 and 0.60). The estimated average correlation coefficient was 0.44 (CI95%: 0.36–0.52; I2 = 87.8%; *Q* = 77.398, *p* < 0.001; Egger’s: *p* = 0.59). Other studies adopted instruments that assess correlated constructs such as alexithymia, social desirability, autism symptoms, and theory of mind (predominance of grade B). The pooled correlation estimate for was 0.38 (CI95%: 0.30–0.46; I2 = 93.8%; *Q* = 194.799, *p* < 0.001; Egger’s: *p* < 0.001) (see Supplementary Material 3.3).

Divergent validity was mainly verified through instruments assessing specific psychiatric symptoms such as hallucination, delirium, hypomania, and systematization (an individual’s ability to develop a system and analyze its variables, considering underlining rules that guide the system’s behavior) (*N* = 4). The values found in these studies ranged from −0.33 and 0.24 and obtained grade A.

Still, in search of evidence of validity with other variables, most studies (*N* = 9) assessed differences between genders; women tended to rate higher in empathy than men, especially in the emotional factor (grade A predominated). Only Gouveia et al. (2012) investigated the EQ’s scores concerning age and education. The authors verified that older age was accompanied by a decline in the EQ’s emotional and social subscales. Education was associated with more frequent expressions of empathy in the instrument’s cognitive, emotional, and social subscales. Only one study tested and verified the instrument’s invariance regarding gender (Senese et al., 2016). Predictive validity/responsiveness was not investigated, revealing a gap in the literature.

The exploratory factor analyses presented models with a varied number of factors, which, however, did not explain the significant percentage of data variance (<47.4%) (Hair et al., 2009). The well-established models proposed by Lawrence et al. (2004; 3 factors: Cognitive Empathy, Emotional Reactivity and Social Skills −28 items), and Muncer and Ling (2006; 3 factors: Cognitive Empathy, Emotional Reactivity, and Social Skills −15 items) were the ones most frequently tested in confirmatory analyses. The results signaled goodness of fit problems for most of the studies; only one-third was rated A in this regard. These two models’ unidimensionality was also tested, presenting contradictory results, while the one-factor model for the 40- and 60-item versions was considered inadequate by the three studies assessing it.

Alternative three-factor models were tested for the 40- and 60-item versions and did not found satisfactory goodness of fit indexes. Other models with a varied number of items and factors were also analyzed (*n* = 8). Those that obtained grade A included: 29-item/4-factor model (Zhang et al., 2018), 23-item/with one or 3-factor model (Redondo and Herrero-Fernández, 2018), 25-item/2-factor model, 15-item/one-factor model (Zhao et al., 2018), and 14-item/one-factor model (Kosonogov, 2014).

Regarding the instrument’s format, Wright and Skagerberg (2012), Senese et al. (2016), and Zhao et al. (2018) tested the online format, the psychometric indicators of which were similar to the original version (pencil-and-paper format). However, the invariance between the versions was not objectively tested.

Note that Wright and Skagerberg (2012) tested an alternative version of the EQ-40, rewriting negative statements into positive to test the hypothesis that the original format was syntactically more complex and challenging. They verified that response time was shorter in the alternative format; however, the remaining psychometric findings were not the same as in the original version, so that the authors did not recommend its use.

### Interpersonal Reactivity Index

Ten studies assessed the psychometric properties of the IRI’s original and alternative versions (with 26, 16, and 15 items). The instrument was considered adequate in terms of Respondent burden and Administrative burden, either due to its brevity or ease of application and interpretation; grade A was obtained.

In terms of normative aspects, as previously observed with the EQ, 70% of the studies only presented data concerning the samples’ mean scores and their respective standard deviations (grade B).

As for the Measurement Model criterion, only the studies by Koller and Lamm (2014), and Ingoglia et al. (2016) investigated this criterion. The first study assessed floor and ceiling effects, while the latter reported kurtosis and asymmetry indicators to verify the normality of the data distribution. The remaining (80%) did not perform analysis with this purpose, so that there is a lack of studies analyzing items.

Half of the studies addressing the IRI presented its cross-cultural adaptation into different languages (Spanish, Portuguese from Brazil, French, and Russian), in general presenting adequate methodology to assess face validity.

Reliability was verified through internal consistency and temporal stability. Moderate meta-analytic measures of internal consistency were found for each subscale (Empathic Concern: 0.70 (CI95%: 0.67–0.72), I2 = 87.02%, *Q* = 78.399, *p* < 0.001, Egger’s: *p* = 0.14; Fantasy: 0.78 (CI95%: 0.77–0.80), I2 = 77.75%, *Q* = 49.355, *p* < 0.001, Egger’s: *p* = 0.84; Personal Distress: 0.72 (CI95%: 0.70–0.74), I2 = 81,01%, *Q* = 56.145, *p* < 0.001, Egger’s: *p* = 0.93; Perspective Taking: 0.69 (CI95%: 0.67–0.71), I2 = 79.51%, *Q* = 67.167, *p* < 0.001, Egger’s: *p* = 0.05 see Supplementary Material 4.1). Test-retest reliability (8 to 12 weeks), even though restricted to two studies, presented excellent indexes (>0.76).

Regarding validity, most studies focused on analyzing the scale’s factorial structure, in which various models were tested using exploratory (*N* = 5) and confirmatory factor analyses (*N* = 8). Davis’s (1983) original model (1983; 4 factors – Empathic Concern, Fantasy, Perspective Taking, and Personal Distress) was the most frequently tested model, though controversial, and in general unsatisfactory results were found. Four-factor alternative models were also investigated, with slightly superior results [e.g., Braun et al. (2015) –15 items and Formiga et al. (2015) –26 items]. Unidimensional and bidimensional models (*N* = 3) were assessed and also presented controversial results.

Convergent validity was performed with other three instruments to assess general empathy and instruments measuring correlated constructs such as positive and negative affect, self-esteem, anxiety, aggression, social desirability, social avoidance, emotional fragility, emotional intelligence, gender roles, and sense of identity. The correlations with correlated constructs tended to be higher (estimated average correlation coefficient: 0.45 (CI95%: 0.34–0.56), I2 = 96.17%, *Q* = 482.604, *p* < 0.001, Egger’s: *p* = 0.02) than the correlations with the construct itself [estimated average correlation coefficient: 0.31 (CI95%: 0.22–0.40), I2 = 75.38%, *Q* = 43.065, *p* < 0.001, Egger’s: *p* = 0.99]. The analysis of subgroups, considering each of the subscales individually, presented the following estimated mean values of correlation with other empathy measures: Empathic Concern: 0.46 (CI95%: 0.27–0.66), Fantasy: 0.26 (CI95%: 0.04–0.49), Personal Distress: 0.25 (CI95%: 0.17–0.34) and Perspective Taking: 0.28 (CI95%: 0.16–0.41) and Personal Distress: 0.25 (CI95%: 0.17–0.34) (see Supplementary Material 4.2).

In most cases, validity based on other variables was assessed in terms of gender. However, Gilet et al. (2013) also investigated age differences, reporting that younger individuals tended to be more empathic than older individuals, especially in the Fantasy and Personal Distress subscales. The studies addressing the IRI did not investigate predictive validity or responsiveness. The only alternative to the instrument’s original format (pencil-and-paper) was a computer version addressed by Chrysikou and Thompson (2016), comparing the equivalence between both (not invariance).

Invariance of the IRI model was verified for sex (Koller and Lamm, 2014; Braun et al., 2015; Ingoglia et al., 2016; Lucas-Molina et al., 2017) and age (Koller and Lamm, 2014; Ingoglia et al., 2016).

### Questionnaire of Cognitive and Affective Empathy

The studies addressing the QCAE involved its original proposition (Reniers et al., 2011) and well-conducted cross-cultural adaptations into French, Portuguese (Portugal), and Chinese, except for the fact that they did not use a pilot study to check for face validity (predominance of B grade).

The QCAE was considered a brief instrument; its application takes no more than 15 min. Scoring is manual and easy to interpret, rating the highest in terms of Respondent and Administrative burden quality. In general, these studies involved basic constructs of the Classic Psychometric Theory, while no data concerning standardization and item analysis were reported.

The alpha cumulative value for the total scale was 0.86 (CI95%: 0.86–0.87; I2 = 0%; *Q* = 1.585, *p* = 0.66; Egger’s: *p* = 0.64) and for the subscales it ranged from 0.61 (Peripheral Responsivity: CI95%: 0.56–0.66; I2 = 79.79%; *Q* = 27.311, *p* < 0.001) to 0.87 (Perspective Taking: CI95%: 0.85–0.88; I2 = 76.19%; *Q* = 18.741, *p* = 0.002). For more details see Supplementary Material 5.1. Test-retest reliability (*r* = 0.76) was satisfactory; the latter was only reported by Liang et al. (2019).

Regarding validity, there was a predominance of studies investigating the scale’s factorial structure (*n* = 5). The original study reports a five-factor structure (Perspective Taking, Emotion Contagion, Online Simulation, Peripheral Responsivity, and Proximal Responsivity) and few goodness-of-fit problems (grade A). Later, most studies (75%) confirmed this structure and report adequate indexes (grade A). Some alternative models were investigated, and findings indicate acceptable goodness-of-fit for the QCAE’s first and second-order four-factor structure (Liang et al., 2019), though the instrument’s unidimensionality was not confirmed.

Regarding convergent validity with other empathy measures (*n* = 2 studies: Basic Empathy Scale and IRI), correlation ranged from 0.27 to 0.76 and obtained grade A (according to the criterion established, only one correlation above 0.60 was necessary to obtain the highest grade). Instruments were also used to assess correlated constructs (e.g., aggressiveness, alexithymia, impulsivity, interpersonal competence, psychopathy, and social anhedonia, among others). The coefficients in these studies were moderate, and most were graded. The estimated average correlation coefficient was 0.27 (CI95%: 0.20–0.35), I2 = 92.12%, *Q* = 183.846, *p* < 0.001, Egger’s: *p* = 0.04 – see Supplementary Material 5.2.

Studies addressing known groups analyses (gender) predominated (*N* = 3), reinforcing previous studies indicating that women have greater empathic ability than men. None of the studies addressing the QCAE investigated predictive validity or responsiveness.

One of the studies (Di Girolamo et al., 2017) assessed the equivalence between the pencil-and-paper and online formats, and both presented similar psychometric indexes and measurement invariance. Invariance was also verified for sex (Myszkowski et al., 2017; Liang et al., 2019).

### Active-Empathic Listening Scale

The AELS was proposed in 2006 (Drollinger et al., 2006) for the specific context of the relationship established between seller and customer, but Bodie (2011) proposed its expanded use for interpersonal relationships in general. Even though Bodie (2011) reported that the original items had to be changed and adapted, no information concerning the items analysis was provided, so that the item/instrument bias criterion obtained grade C.

Later, Gearhart and Bodie (2011) expanded the adapted version’s psychometric studies, presenting well-assessed Respondent burden and Administrative burden (grade A). Only Bodie (2011) assessed internal consistency, and the coefficients for the instrument as a whole (>0.86) were considered excellent (grade A).

From the factorial structure perspective, the three-factor model (Sensing, Processing, and Responding) was considered appropriate, specifically for the self-report version (grade A) (Bodie, 2011), which was later confirmed by Gearhart and Bodie (2011) (grade A).

The convergent validity indexes concerning the self-report version indicated that correlations for the correlated constructs (conversational adequacy, interaction implications, social skills; −0.16 to 0.67 – grade A) were more robust than for the general empathy construct (0.15 to 0.44), which were considered moderate (grade B). Only correlations with correlated constructs (conversational adequacy, conversational effectiveness, non-verbal immediacy) were investigated for the other-report version, ranging from 0.15 to 0.75, and considered adequate (grade A). The self-report and other-report versions evidenced invariance of measure.

Studies addressing validity with other variables investigated the relationship between empathy scores and whether an individual is considered a good or poor listener (having an active and emotional interaction or not). Good listeners tended to score higher in empathy. There are no studies addressing the AELS normative data or predictive validity or studies conducting cross-cultural adaptations.

### Toronto Empathy Questionnaire

The Toronto Empathy Questionnaire (TEQ) was addressed by two studies between 2009 and 2019: the study that originally proposed it (Spreng et al., 2009) and the study of its cross-cultural adaptation into Turkish (Totan et al., 2012). The process of developing TEQ was adequately described, but no pilot test was reported. A pilot test was implemented during its cross-cultural adaptation, impeding the Item/instrument bias criterion from achieving the maximum grade. On the other hand, due to the instrument’s ease of use and application, the Respondent burden and Administrative burden criteria were assessed and obtained grade A.

The TEQ’s reliability was assessed using internal consistency (α = 0.79 to 0.87; predominance of grade A) and temporal stability (0,73; grade B), which were adequate. In terms of factorial structure, Spreng et al. (2009) conducted two exploratory analyses, and a unidimensional structure was found in both, with factor loadings above 0.37 (grade B). Totan et al. (2012) replicated the TEQ’s unifactorial structure but found problems in three specific items, which led them to retest the model after excluding these items. Both the 16-item and 13-item versions appeared satisfactory in the confirmatory analysis, and the shortest version was recommended.

The TEQ’s convergent validity was verified by comparing other instruments measuring empathy and instruments assessing correlated constructs such as autism, ability to understand the mental states of others, and interpersonal perception. As expected, most correlations between TEQ and other instruments assessing empathy were higher (0.29 to 0.80; grade A) than correlations with correlated constructs (−0.33 to 0.35; grade B).

Finally, other evidence of validity was analyzed, having the gender as a reference, and showed that women scored higher than men. The TEQ studies did not investigate predictive validity or responsiveness and did not report alternative formats or transcultural adaptations.

### Empathy Assessment Index

Gerdes et al. (2011) originally proposed the EAI, and Lietz et al. (2011) later assessed its psychometric properties. The authors described the process of instrument development and the theoretical conceptualization of each of the five factors composing it (Affective Response, Perspective Taking, Self-Awareness, Emotion Regulation and Empathic Attitudes); Grade A was granted to the Item/instrument bias criterion.

Like the remaining instruments presented thus far, the EAI was also considered easy to apply, and therefore, the Respondent burden and Administrative burden were rated with the highest grade. Its precision coefficients ranged from 0.30 to 0.83, and temporal stability ranged from 0.59 to 0.85; the retest was applied with a 1-week interval (grade B).

The original study reports that the exploratory factor analysis indicated a 34-item and 6-factor structure (Empathetic Attitudes, Affective Response -happy, Perspective Taking, Affective Response -sad, Perspective Taking-Affective Response and Emotion Regulation), which explained 43.19% of the variance of data (grade B). Later, based on literature reviews and feedback provided by specific community groups and experts in empathy, Lietz et al. (2011) performed factor analyses for a new 48-item version, concluding that the model presenting the best goodness of fit was composed of 17 items and five factors (Affective Response, Perspective Taking, Self-Awareness, Emotion Regulation and Empathic Attitudes) (grade A).

Gerdes et al. (2011) verified the convergent validity of the 34-item version, comparing it with the IRI and the coefficients ranged from 0.48 to 0.75; grade A was obtained. Lietz et al. (2011) investigated convergent validity by comparing the EAI with correlated constructs, such as attention and cognitive emotion regulation. As expected, moderate coefficients were found (−0.40 to 0.51; grade B).

Lietz et al. (2011) verified the EAI’s validity concerning the different sociodemographic variables. The results suggest differences concerning race (Afro- and Latin-Americans tended to present greater empathetic behavior than Caucasians); educational background (social workers presented greater empathy than individuals from the criminal justice, sociology, education, or nursing fields); and the family of origin’s socioeconomic status (poor/working-class individuals presented greater empathetic behavior than middle/high-class individuals). The studies addressing the EAI did not address predictive validity or responsiveness nor reported alternative formats.

### Affective and Cognitive Measure of Empathy

Affective and cognitive measure of empathy (ACME) was proposed by Vachon and Lynam (2016), and later, new validity evidence was presented by Murphy et al. (2018). Vachon and Lynam did not report the procedures concerning the instrument’s development so that the Item/instrument bias criterion obtained grade C. However, the instrument presented the characteristics necessary to receive grade A in the Respondent burden and Administrative burden criteria. Note that Vachon and Lynam (2016) study obtained grade B in the Norms and standard values criteria because only the means and standard deviations of each of the instrument’s scales were reported according to sex and race for the entire sample.

Its reliability was only investigated through the internal consistency method (>0.85; Vachon and Lynam, 2016), indicating a gap concerning temporal stability indicators.

Investigations related to the instrument’s factorial structure and convergent validity were found. Vachon and Lynam (2016) suggested a three-factor structure (Cognitive Empathy, Affective Resonance and Affective Dissonance) with satisfactory goodness-of-fit indexes (grade A) and invariance between genders. However, Murphy et al. (2018) were unable to replicate this model and obtained unsatisfactory goodness-of-fit indexes. Hence, they proposed a five-factor model (two factors based on the items’ polarity – positive and negative items, in addition to Cognitive Empathy, Affective Resonance and Affective Dissonance factors), with presented criteria that obtained grade A.

Convergent validity was verified in relation to IRI (−0.24 to 0.80) and the Basic Empathy Scale (0.40 to 0.65); these criteria obtained grade A. The results indicated grade A for both studies regarding the indexes concerning correlated constructs, such as aggressive behavior, externalizing disorders, and personality pathologies (−0.83 to 0.77).

### Remaining Instruments

Other 16 instruments were analyzed by single studies. Seven of these intended to propose new instruments, five intended to obtain additional validity evidence, and four conducted a cross-cultural adaptation of existing instruments.

Except for the MET, all the instruments obtained grade A in the Respondent burden and Administrative burden criteria because they were brief, well-accepted, easy to apply, score and interpret. MET obtained grade B in both items because it is composed of 80 items and its application/scoring requires specific software.

Analysis of the new instruments showed no specific normative indicators were reported for any of them (Norms, standard values, grade B). Reliability verified through internal consistency was investigated in 85.7% of the studies and, in general, presented satisfactory results (grade B). Temporal stability was verified in only 28.6% of the studies and presented positive results (grades A and B).

As for existing instruments, there is a lack of normative data (data reported by 33.3% of the studies were restricted to mean and standard deviation of the total score). Nonetheless, as verified in the studies previously presented, no specific comparison indicators were reported between groups (e.g., T score or percentile).

Note that only two studies addressing these new instruments (Segal et al., 2013 – Interpersonal and Social Empathy Index and Batchelder et al., 2017 – Empathy Components Questionnaire) reported information concerning how the instruments were developed and obtained grade A. Convergent (*n* = 7), factor (*n* = 6), discriminant (*n* = 5), and predictive (*n* = 2) analyses were performed to investigate the instruments’ validity. Regarding the factorial structure, both the instruments proposed before 2009 and those proposed after 2009 obtained grade B, showing that this group of instruments’ factorial structures was confirmed with a few goodness-of-fit problems.

In general, the quality of the results concerning convergent validity was considered moderate (grade B). Correlations with other instruments measuring empathy were similar to the correlations found with instruments measuring correlated constructs. Validity studies with other variables were also restricted to the investigation of gender, corroborating the findings reported in the literature; that is, women tend to be more empathic than men. Hollar (2017) expanded the variables of interest (age and ethnicity) but obtained no satisfactory results.

Among this group of studies, Oceja Fernández et al. (2009) investigated predictive validity concerning the Vicarious Experience Scale; the Sympathy and Vicarious Distress subscales did not present satisfactory indexes for the prediction of elicited empathy and personal anguish. Batchelder et al. (2017), in turn, report the predictive ability of the Empathy Components Questionnaire concerning the scores obtained in the Social Interests Index (grade B).

Regarding this group of instruments, note that the Pictorial Empathy Test (Lindeman et al., 2018) differs from the remaining. It presents higher ecological validity because it is composed of images of people, while the Single Item Trait Empathy Scale stands out because it is composed of a single item. In general, both presented satisfactory psychometric properties.

As for cross-cultural studies, in general, face validity procedures were in line with the guidelines recommended by Beaton et al. (2000), and most (75%) obtained grade A. These studies’ psychometric properties were considered satisfactory, while the Culture/language adaptations item obtained grade B.

The instruments’ reliability (internal consistency and temporal stability) was considered acceptable in most studies (grades A and B). However, the cross-cultural study addressing the MET obtained indexes below the expected for the instrument’s cognitive factor, even after decreasing the scale’s number of items.

Six studies investigated the instruments’ factorial structure. The results showed acceptable indexes for the Measure of Empathy and Sympathy, Multidimensional Emotional Empathy Scale, Basic Empathy Scale, The Mexican Empathy Scale, Positive Empathy Scale, and Cognitive, Affective, and Somatic Empathy Scales.

Among this set of studies, Park et al. (2019) conducted a cross-cultural adaptation of the Cognitive, Affective, and Somatic Empathy Scales. This instrument presents a specific scale to assess somatic empathy, which, according to the authors, can be defined as a tendency to imitate and automatically synchronize other peoples’ facial expressions, vocalizations, behaviors, and movements. Only this instrument presented this measure. In general, its psychometric qualities were considered satisfactory.

## Discussion

This review compiled the psychometric findings of 23 instruments available in the literature to assess empathy in the last 10 years. In general, the findings concerning the existent instruments [reliability generalization meta-analyses (Cronbach’s alpha) with values between 0.61 and 0.86] reinforced previous indicators of adequate reliability (e.g., EQ: Baron-Cohen and Wheelwright, 2004; Lawrence et al., 2004; IRI: Davis, 1980; Cliffordson, 2002; AELS: Drollinger et al., 2006; MxES: Díaz-Loving et al., 1986; MDEES: Caruso and Mayer, 1998; BES: Jolliffe and Farrington, 2006; EI: Falcone et al., 2008; MET: Dziobek et al., 2008).

On the other hand, the results indicated problems concerning convergent validity and factorial structure, making little progress in the solution and discussion of these impasses, e.g., a low to moderate correlation was found, especially between the EQ, IRI and QCAE and other instruments assessing the empathy construct (meta-analytic measures of correlation between 0.31 and 0.44), while similar or higher values were found when correlating these with different correlated constructs (meta-analytic measures of correlation between 0.27 and 0.45), indicating that the instruments’ clinical validity was greater than theoretical validity. Studies published before the period addressed in this review also indicated these limits concerning convergent validity (e.g., EQ vs. IRI: Lawrence et al., 2004; De Corte et al., 2007; IRI vs. Hogan Empathy Scale: Davis, 1983; AELS vs. IRI: Drollinger et al., 2006; BES vs. IRI: Jolliffe and Farrington, 2006; MET vs. IRI: Dziobek et al., 2008) and factorial structure (e.g., EQ: Lawrence et al., 2004; Muncer and Ling, 2006; IRI: Siu and Shek, 2005; De Corte et al., 2007; BES: Jolliffe and Farrington, 2006; EI: Falcone et al., 2008).

It is important to note that most of the instruments analyzed here did not reach a consensus regarding the best factorial structure, considering that various models were tested. The results concerning the goodness of fit suggest problems related to both the base model (Comparative Fit Index and Tucker Lewis Index below the expected) and population covariance (Root Mean Square Error of Approximation above the expected) (Bentler, 1990; Hu and Bentler, 1999; Thompson, 2004). These divergences possibly reflect on the analyses of convergent validity with the different instruments measuring empathy, most of which obtained values within moderate limits.

We believe that controversies concerning the multidimensional nature of empathy (Murphy et al., 2018) reflect on the analyses, especially when the target instruments’ subscales are more specifically analyzed. Murphy et al. (2018) widely discuss these aspects and note a lack of consensus regarding the empathy construct and how different authors adopt such a concept when developing instruments. These authors note there is greater consensus regarding the presence of affective and cognitive components; however, the analyses of the bifactor models assessed here (e.g., concerning the IRI) also failed to present satisfactory factor indexes. Given this lack of consensus, Surguladze and Bergen-Cico (2020) stress the need to reconsider and discuss this construct, considering its different dimensions and directly and indirectly related mechanisms.

In addition to what Murphy et al. (2018) state regarding lack of consensus, this review’s findings indicate that some studies do not specify the conceptual model of empathy that grounded the development of the instruments and which would theoretically ground the empirical analysis of the instruments’ internal structure, especially in second-order more complex models and with a varied number of factors. It was the case of both new instruments, proposed during the period covered by this review (for example: QCAE: Reniers et al., 2011; QoE: Miguel et al., 2018; TEQ: Spreng et al., 2009), and older instruments (EQ: Baron-Cohen and Wheelwright, 2004; IRI: Davis, 1983; MxES: Díaz-Loving et al., 1986; EI: Falcone et al., 2008). A lack of theoretical models to properly ground the empathy construct and its dimensions possibly explains the restrictions concerning structural validity and lack of convergence between the different instruments.

Regarding the statistical techniques used to investigate the instruments’ structures, Marsh (2018) highlight that newer models, such as the Structural Equations Models (Gefen et al., 2000), can more deeply capture the complexity of the empathy construct and also resolve a series of problems encountered based on the CFA approach (e.g., restricted factor loadings) (Marsh, 2018). Nonetheless, most of the studies opted for adopting confirmatory and exploratory factor analyses so that future studies are needed to invest in these technologies and techniques of analysis. Note that studies based on the Item Response Theory (Pasquali, 2020) can contribute to this impasse, considering that the studies addressed here attempted to improve the factor model by removing specific items.

On the other hand, the construct’s clinical/empirical validity seems to be unanimous. Even though the studies were conducted with non-clinical samples, associations with different correlated constructs are adequate and reinforce the relationship of empathy with different psychopathological and behavioral indicators (e.g., autism: Komeda et al., 2019, Post-traumatic stress disorder: Feldman et al., 2019, and Borderline personality disorder: Flasbeck et al., 2019). However, for these instruments to be used in a clinical setting, aspects related to predictive evidence, which remain scarce, need to be explored. In this context, normative studies, which were not the target of the psychometric studies addressed here, are also needed.

Cross-cultural studies were an important focus of interest among researchers within this topic. These are relevant studies because they enable generating and/or reinforcing psychological theories that take the cultural context into account (Gomes et al., 2018). Additionally, these studies enable applying the same instrument among different individuals belonging to different contexts and facilitate understanding the similarities and characteristics these groups share (Borsa et al., 2012), which is essential, especially in clinical research.

In general, the results of cross-sectional studies addressing instruments reported psychometric qualities comparable to the original versions, though cultural invariance was not assessed for any of the target instruments. Investigating invariance between different cultural groups answering an instrument is essential to identify whether there are significant differences between scores, and if that is the case, verify whether differences are related to actual differences at a latent trait level or the instruments’ parameters are not equivalent (Damásio et al., 2016).

Regarding new instruments, note that the same few added in terms of Respondent burden and Administrative burden, considering that these aspects, except for the MET, obtained grade A, though were little discriminant. These instruments also innovated little in terms of format and structure. Most were based on self-reported items rated on a Likert scale. The studies also do not seem to overcome the critical points mentioned earlier in psychometric terms.

Interpersonal reactivity index, and more recently, EQ, have been widely used in different clinical studies and applied in different target populations (e.g., Feeser et al., 2015; Fitriyah et al., 2020). However, despite their popularity, they present weaknesses concerning structural validity and limitations regarding responsiveness, standardization, and bias.

The conclusion is that despite the diversity of the instruments available to assess empathy and many associated psychometric studies, limitations stand out, especially in terms of validity. Hence, as noted by previous reviews that evaluated specific instruments of empathy and/or their performance in specific populations (Hemmerdinger et al., 2007; Yu and Kirk, 2009; Hong and Han, 2020), no instrument can be currently appointed as the gold standard.

Therefore, this field of study needs to advance in conceptual and theoretical terms. Such an advance will enable the establishment of more robust models to be empirically reproduced by the instruments. Additionally, problems with the internal structure of various instruments can be minimized or resolved using more sophisticated techniques based on the analysis and refinement of items. Normative and predictive studies can improve the validity of evidence of existing studies, favoring greater clinical applicability. Complementary studies of invariance, testing the effect of cultures, and alternative forms of application (especially those using technological resources, such as online and computer applications) are desirable and can expand the reach of instruments. Regarding the proposition of new instruments, there seems to remain a need for instruments with alternative formats to minimize response bias, especially social desirability, a recurrent problem in self-report instruments.

## Data Availability Statement

The raw data supporting the conclusions of this article will be made available by the authors, without undue reservation.

## Author Contributions

Both authors listed have made a substantial, direct, and intellectual contribution to the work, and approved it for publication.

## Conflict of Interest

The authors declare that the research was conducted in the absence of any commercial or financial relationships that could be construed as a potential conflict of interest.

## Publisher’s Note

All claims expressed in this article are solely those of the authors and do not necessarily represent those of their affiliated organizations, or those of the publisher, the editors and the reviewers. Any product that may be evaluated in this article, or claim that may be made by its manufacturer, is not guaranteed or endorsed by the publisher.
