# Supplementary material for: Empathy: Assessment Instruments and Psychometric Quality – A Systematic Literature Review With a Meta-Analysis of the Past Ten Years
Source: Front Psychol. 2021 Nov 24;12:781346. doi: 10.3389/fpsyg.2021.781346 (PMC8653810; doi:10.3389/fpsyg.2021.781346)
Supplement: Supplementary file 1 [file Data_Sheet_1.docx]

Supplementary Material

| **Supplementary Material 1 -** Criteria for assessing psychometric properties (adapted from Andresen, 2000) | | |
| --- | --- | --- |
| **Properties** |  | **Criteria for Grades** |
| **Norms/standard values** |  | Published data (or public-domain data) are available for:  A = both general population AND clinical groups  B = either general population OR clinical groups  C = neither general nor clinical groups |
| **Measurement model** |  | The measure has scales/measure with 20% of clinical groups grouped at scoring extremes:  A = no problems  B = few or marginal problems  C = substantial skewing of scales/measure |
| **Item/Instrument Bias** |  | Qualitative:  A = face validity tested, with suitability report. Non-existent bias and acceptability  B = there is adequate face validity to support low bias  C = bias is evident  Statistical evaluation  A = Rasch analysis is good  B = factorial analysis is good OR Rasch analysis shows some problems  C = inadequate statistical analysis |
| **Respondent Burden** |  | A = brief (<15min) and acceptability high for target population  B = either longer (but appropriately so) or some reported problems of acceptability  C = both length and acceptability are problematic for target population |
| **Administrative Burden** |  | A = manual scoring and the resulting metric is relevant and interpretable for researchers, clinicians and target population  B = computer scoring and more obscure interpretation  C = costly and/or complex scoring and/or interpretation |
| **Reliability** |  | Retest: Kappa correlation coeficiente  A ≥ 0.75  B > 0.40, < 0.75  C ≤ 0.40  Internal consistency: coefficient alpha  A ≥ 0.80  B > 0.70, < 0.80  C ≤ 0.70  Respondent's answer: intraclass correlation coeficient  A ≥ 0.75  B > 0.40, < 0.75  C ≤ 0.40 |
| **Validity** |  | Convergent Validity / Multitrait-multimethod matrix  A ≥ 0.60  B > 0.30, < 0.60  C ≤ 0.30  *Note*: the attribution of grades prioritized the following hierarchy of correlations: i) Total score of the target instrument x Total score of the reference instrument; ii) Total score of the target instrument x subscales of the reference instrument; iii) Subscales of the target instrument x subscales of the reference instrument. To obtain the highest criterion, it was necessary to have only one correlation within the range.  Known groups - Differences by average or percentage  Patient-centered and/or clinical criteria for change are:  A = strong, expected direction, supported by patient or clinical evidence  B = moderate or conflicting evidence  C = weak or based solely on statistical significance  Factorial Structure  A = confirmed  B = few problems  C = weak or unconfirmed  *Note*: Studies with at least 3 indexes* were evaluated, and the classification was performed as follows:  A = 3 good indexes  B = 2 good indexes  C = 1 or no good index  *Studies with less than 3 indexes can obtain a maximum of grade B, if they have 2 good indexes, and studies with only one index are automatically evaluated with grade C. |
| **Responsiveness/ Predictive validity** |  | A= strong, expected direction, supported by patient or clinical evidence  B = moderate or conflicting evidence  C = weak or based solely on statistical significance |
| **Alternative/acessible forms** |  | A = appropriate or varied modes are available and tested; accommodations are available  B = some adaptations OR tests for different formats  C = without adaptations OR information related to different formats |
| **Culture language adaptation** |  | A = evidence of testing and broad applicability for cultural subgroups, and interpretations are well described  B = evidence of translations or testing with subgroups, some problems  C = no evidence of testing or applicability to groups based on culture or language |

| **Supplementary Material 2 -** Psychometric properties of the various instruments, according to the studies analyzed (n = 50**)** | | | | | |  |
| --- | --- | --- | --- | --- | --- | --- |
| Instrument | Study/year | Objective | Reliability | Validity Evidences | | |
|  |  |  | **α/TR** |  |  |  |
| Empathy Quotient (EQ) | Kim & Lee (2010)  EQ-40 | To adapt the EQ-40 to the korean context and to establish its psychometric properties. In addition, it aims to evaluate the factorial structure of reduced versions, proposed by Lawrence et al (EQ-28, 2004) and Muncer and Ling (EQ-15, 2006) | Cronbach’s alphas:  **EQ-40 - 3 Factors Model:** α=0.78  T.R. (4w):  **EQ-40 - 3 Factors Model:** r=0.84 | Kurtosis=0.15/ Skewness= 0.11  CFA:  **EQ-40 - 3 Factors Model (Cognitive Empathy, Emotional Reactivity and Social Skills):** GFI=0.75; CFI=0.83; RMSEA=0.09  **EQ-28 - 1 Factor Model:** GFI=0.81; CFI=0.87; RMSEA=0.087  **EQ-28 - Lawrence et al - 3 Factors Model (Cognitive Empathy, Emotional Reactivity and Social Skills):** GFI=0.87; CFI=0.92; RMSEA=0.056  **EQ-15 - Muncer & Ling - 3 Factors Model (Cognitive Empathy, Emotional Reactivity and Social Skills):** GFI=0.94; CFI=0.93; RMSEA=0.056  Convergent:  **EQ-40 total/SS x IRI SS:** r= -0.35 to 0.34  Known groups (Gender):  **EQ-40 - women=men:** p=0.22  **SS-Cognitive empathy - women=men:** p=0.71  **SS-Emotional Reactivity - women>men:** p=0.001  **SS-Social Skills - women<men:** p<0.001 | | |
|  | Preti et al. (2011)  EQ-60 | To evaluate the cross-cultural validity of EQ-60 for the Italian population | Cronbach’s alphas:  **EQ-60 - 3 Factors Model:** α=0.79  T.R. (4w):  **EQ-60 - 3 Factors Model:** r=0.76 to 0.85 | CFA:  **EQ-60 - 3 Factors Model (Social Skills, Cognitive Empathy, Emotional Reactivity):** x²=618.69(p<0.01); GFI=0.85; RMSEA=0.06; SRMSR=0.07  Convergent:  **EQ-60 SS x Toronto Alexithymia Scale (related construct):** r= -0.38 to 0.25  **EQ-60 SS x Marlowe-Crowne Social Desirability Scale SS-Positive Attribution (related construct):** r=0.21 to 0.25  **EQ-60 SS x Marlowe-Crowne social desirability scale SS-Denial (related construct):** r=0.16 to 0.35  Discriminant:  **EQ-60 SS x The Peters et al. Delusions Inventory:** r= -0.31  **EQ-60 SS x The Revised Launay-Slade Hallucination Scale:** r= -0.32  **EQ-60 SS x The Hypomanic Personality Scale:** r= -0.16 to 0.23  Known groups (Gender):  **EQ-60 - women>men:** p=0.002, cohen’s d=0.39 | | |
|  | Rodrigues et al. (2011)  EQ-22 | To validate the short version of the EQ-22 scale for the Portuguese population | Cronbach’s alphas:  **EQ-22 - 4 Factors Model:** α=0.85  **SS-Cognitive Empathy:** α=0.80  **SS-Emotional Reactivity:** α=0.71  **SS-Social Skills:** α=0.70  **SS-Empathic Difficulties:** α=0.66 | EFA:  **4 Factors Model (Cognitive Empathy, Emotional Reactivity, Social Skills, Empathic Difficulties)** = 47.49% of variance, Factor loadings > 0.40  Discriminant  **EQ Total x Systemizing Quotient:** r=0.24  **EQ SS x Systemizing Quotient:** r=0.11-0.20 | | |
|  | Gouveia et al. (2012)  EQ-15 | To examine the psychometric properties of EQ-15 in Brazilian context | Cronbach’s alphas:  **SS-Emotional:** α=0.45  **SS-Social:** α=0.50  **SS-Cognitive:** α=0.72  Composite reliability**:**  **SS-Emotional:** 0.43  **SS-Social:** 0.66  **SS-Cognitive:** 0.68 | CFA:  **EQ-15 - 1 Factor Model:** χ²=229.56(p<0.001); AGFI=0.85; CFI=0.73; RMSEA=0.08  **EQ-15 - Muncer & Ling - 3 Factors Model (Cognition, Emotional, Social):** χ²=193.37(p<0.001); AGFI=0.86; CFI=0.79; RMSEA=0.07  Known groups:  *Age:*  **SS-Emotional:** r= -0.16, p<0.05  **SS-Social:** r= -0.25, p<0.01  *Gender:*  **SS-Cognitive - women>men:** r=0.14, p<0.05  **SS-Emotional - women>men:** r=0.21, p<0.01  **SS-Social - women=men:** r=0.09, p>0.05  *Level education:*  **SS-Cognitive:** r=0.28, p<0.05  **SS-Emotional:** r=0.19, p<0.01  **SS-Social:** r=0.16, p<0.05 | | |
|  | Wright & Skagerberg (2012)  EQ-40 | To investigate on the EQ-40, the processing of positive word items (unchanged version = original version with some negative items x changed version = positive items with sentences.), explore people's accuracy in judging their own levels of empathy | Cronbach’s alphas:  **EQ-40 Unchanged version:** α=0.87  **EQ-40 Changed version:** α=0.86 | Comparison of Response Times for Non-Critical Items:  **Unchanged version=Changed Versions:** NS  Comparison between response times for critical items:  **Unchanged version<Changed Versions:** p<.001  Convergent:  **EQ-40 Changed Version x Self-assessed Empathizing:** r=0.55 to 0.59  **EQ-40 Changed Version x Autism-Spectrum Quotient Test (related construct):** r= -0.59  Discriminant:  **EQ-40 x Systemizing Quotient:** r=0.22  **EQ-40 x Self-assessed Systemizing:** r=0.04  Known groups (Gender):  **EQ-40 - women>men:** p<0.001 | | |
|  | Kosonogov (2014)  EQ-60 | To investigate the psychometric properties of EQ-60 for the Russian population and propose brief versions of the instrument | Cronbach’s alphas:  **EQ-60 - 1 Factor Model:** α=0.85  **EQ-29 - 3 Factors Model:** α=0.86-0.88  **EQ-28 - Lawrence et al model - 3 factors:** α=0.54-0.86  **EQ-21 - 3 Factors Model:** α=0.63-0.85  **EQ-15 - 3 Factors Model:** α=0.61-0.75  **EQ-15 - Muncer & Ling model - 3 Factors Model:** α=0.55-0.72  T.R. (2w):  **EQ-60 1 Factor Model:** r=0.94 | Kurtosis=0.11/ Skewness=0.18  EFA:  KMO=0.80; Bartlett test of sphericity (p=0.001)  **3 factors (Cognitive, Emotional, Social)** = 32.33% of variance, Factor loadings ≥ 0.41  CFA:  **EQ-60 - 1 Factor Model:** χ²=1531.3; RMSEA=0.09; GFI=0.68; CFI=0.59  **EQ-29 - EFA 3 Factors Model:** χ²=681.6; RMSEA=0.06; GFI=0.82; CFI=0.80  **EQ-28 - Lawrence et al. - 3 Factors Model:** χ²=706.5; RMSEA=0.07; GFI=0.80; CFI=0.76  **EQ-21 (7 items with the highest loads for each of the 3 factors):** χ²=353.3; RMSEA=0.06; GFI=0.86; CFI=0.83  **EQ-15 (5 items with the highest loads for each of the 3 factors):** χ²=175.3; RMSEA=0.08; GFI=0.90; CFI=0.83  **EQ-15 - Muncer & Ling -3 Factors Model:** χ²=162.2; RMSEA=0.06; GFI=0.91; CFI=0.84  **EQ-14 - 1 Factor:** χ²=171.4; RMSEA=0.08; GFI=0.90; CFI=0.90  Convergent:  **EQ-60 x** **Questionnaire Measure of Emotional Empathy:** r=0.34  **EQ-60 x** **Quotient of Empathic Abilities:** r=0.48  Known groups (Gender):  **EQ-40 - women>men:** p<0.05 | | |
|  | Senese et al. (2016)  EQ-40 | To investigate the psychometric properties of EQ-40 for the Italian population and to compare the factorial structure of the 28-item (Lawrence et al., 2004) and 15-item (Muncer & Ling, 2006) versions of the EQ scale | Cronbach’s alphas:  **EQ-15 - 3 Factors Model:** α=0.78  **SS-Cognitive Empathy:** α=0.74  **SS-Emotional Reactivity:** α=0.73  **SS-Social Skills:** α=0.68 | CFA:  **EQ-40 - 1 Factor Model:** χ²=2371.4; CFI=0.65; RMSEA=0.07  **EQ-28 - 1 Factor Model:** χ²=5982.4; CFI=0.95; RMSEA=0.06  **EQ-28 - Lawrence et al - 3 Factors Model (Cognitive Empathy, Emotional Reactivity, Social Skills):** χ²=3279.6; CFI=0.98; RMSEA=0.03  **EQ-15 - 1 Factor:** χ²=1393.2; CFI=0.81; RMSEA=0.11  **EQ-15 - Muncer & Ling - 3 Factors Model (Cognitive Empathy, Emotional Reactivity, Social Skills):** χ²=3279.6; CFI=0.98; RMSEA=0.03  Convergent:  **EQ-15 x IRI SS:** r=0.04 to 0.49  **EQ-15 – IRI SS-Cognitive Empathy:** r=0.06 to 0.25  **EQ-15 – IRI SS-Emotional Reactivity:** r=0.17 to 0.62  **EQ-15 – IRI SE-Social Skills:** r= -0.23 to 0.09  **EQ-15 x Toronto Alexithymia Scale (related construct):** r= -0.31  **EQ-15 SS x Toronto Alexithymia Scale (related construct):** r= -0.36 to -0.13  Discriminant:  **EQ-15 x Hypomania/Mania Symptom Checklist-32:** r= -0.03 (NS)  **EQ-15 SS x Hypomania/Mania Symptom Checklist-32:** r= -0.06 to 0.07 (NS)  Measure invariance (*Gender):*  **Model 1 (equality of covariance matrices across groups):** MLχ2(120)=309.5, p<0.001, SBχ2(120)=161.1, p=0.007, CFI=0.99, RMSEA=0.033  Known groups (Gender):  **EQ-15 SS-Empathic reactivity:** women>men: p<0.001  **EQ-15 SS-Cognitive empathy:** NS  **EQ-15 SS-Social Skills:** NS | | |
|  | Kose et al. (2018)  EQ-60 | To examine the validity, reliability, and factor structure of the EQ-60 in a Turkish sample | Cronbach’s alphas:  α=0.76  T.R. (2w) n=136:  r=0.95 | CFA:  KMO=0.797; Bartlett test of sphericity (p<0.001)  **3 factors (Cognitive, Emotional, Social):** 25.3% variance; 11 items, Factor loadings < 0.30  Convergent:  **EQ-60 x Marlowe-Crowne Social Desirability Scale-33-item (related construct):** r=0.30  **EQ-60 x Marlowe-Crowne Social Desirability Scale-13-item (related construct):** r=0.27  Known groups (Gender):  **EQ-60 - women>men:** p=0.001 | | |
|  | Redondo & Herrero-Fernández (2018)  EQ-60 | To examine the reliability, validity and factor structure of the EQ-28 for the Spanish population | ---- | CFA:  **EQ-28 - Lawrence et al. - 3 Factors Model:** χ²/df=3.30; GFI=0.92; CFI=0.91; NNFI=0.90; RMSEA=0.07  **EQ-23 - 3 Factors Model (items with a factor load > 0.20):** χ²/df=2.76; GFI=0.95; CFI=0.95; NNFI=0.95; RMSEA=0.06  **EQ-23 - 1 Factor Model (items with a factor load > 0.20):** χ²/df=4.0; GFI=0.94; CFI=0.91; NNFI=0.91; RMSEA=0.08  **EQ-15 - Kim & Lee - 3 Factors Model:** χ²/df=4.59; GFI=0.92; CFI=0.85; NNFI=0.83; RMSEA=0.09  **EQ-15 - 1 Factor Model:** χ²/df=4.0; GFI=0.94; CFI=0.91; NNFI=0.91; RMSEA=0.08  Convergent:  **EQ-28 SS x IRI SS-Perspective Taking:** r=0.26 to 0.37  **EQ-28 x IRI-SS-Perspective Taking:** r=0.42  **EQ-28 SS x Reading the Mind in the Eyes (related construct):** r=0.14 to 0.19  **EQ-28 x Reading the Mind in the Eyes (related construct):** r=0.20  **EQ-28 SS x Toronto Alexithymia Scale (related construct):** r= -0.53 to -0.30  **EQ-28 total x Toronto Alexithymia Scale (related construct):** r= -0.50  Known groups (Gender):  **SS-Emotional Reactivity - women>men:** p<0.001, d=-0.56  **SS-Cognitive Empathy - women x men:** NS  **SS-Social Skills - women x men:** NS | | |
|  | Zhang et al. (2018)  EQ-60 | To adapt and study a complete Chinese version of the EQ-60 scale and to assess its reliability and validity among Chinese minority college students | Cronbach’s alphas:  **EQ-29 - 4 Factors Model:** α=0.82  **SS-Cognitive Empathy:** α=0.89  **SS-Emotional Empathy:** α=0.71  **SS-Social-consciousness**: α=0.79  **SS-Social Skills:** α=0.75  T.R. (1w) n=50:  **EQ-29 total:** r=0.90  **EQ-29 SS:** r=0.72-0.94 | CFA:  **EQ-29 - 4 factors (Cognitive Empathy, Emotional Empathy, Social-consciousness, Social Skills):** χ²/df=2.51 <5; GFI=0.94; AGFI=0.92; IFI=0.92; CFI=0.91; TLI=0.90; RMSEA=0.04  Known groups (Gender):  **EQ-29 - women>men:** p<0.001  **SS-Self-consciousness - women>men:** p<0.001  **SS-Cognitive Empathy - women>men:** p=0.092  **SS-Social Skills - women>men:** p<0.001  **SS-Emotional empathy - women>men:** p<0.001 | | |
|  | Zhao et al. (2018)  EQ-60 | To validate a simplified full-size Chinese version (60 items, with 40 and 15 items) of EQ in Mainland China | Cronbach’s alphas:  **EQ-40 - 1 Factor Model:** α=0.86  **EQ-15 - Modified Guan et al. - 1 Factor Model:** α=0.86  **EQ-40 - Lawrence et al. - 3 Factors Model:** α=0.57 to 0.87  **EQ-22 - Wakabayashi et al. - 1 Factor Model:** α=0.86  **EQ-15 - Muncer & Ling - 3 Factors Model:** α=0.55 to 0.78  **EQ-26 - Alisson et al. - 2 Factors Model:** α=0.74 to 0.80  **EQ-15 - Guan et al. - 1 Factor Model:** α=0.86  T.R. (2w) n=35:  **EQ-40:** ICC=0.82  **EQ-15:** ICC=0.68 | CFA:  **EQ-40 - 1 Factor Model:** χ²=3243.12; CFI=0.73; TLI=0.71; RMSEA=0.08; WRMR=2.18  **EQ-15 - Modified Guan et al. - 1 Factor Model:** χ²=359.16 CFI=0.95; TLI=0.95; RMSEA=0.07; WRMR=1.31  **EQ-40 - Lawrence et al. - 3 Factors Model:** χ²=1588.28; CFI=0.84; TLI=0.83; RMSEA=0.08; WRMR=1.93  **EQ-22 -** **Wakabayashi et al. - 1 Factor Model:** χ²=1200.55; CFI=0.86; TLI=0.84; RMSEA=0.09; WRMR=1.93  **EQ-15 - Muncer & Ling - 3 Factors Model:** χ²=360.04; CFI=0.86; TLI=0.84; RMSEA=0.07; WRMR=1.93  **EQ-26 - Alisson et al. - 2 Factors Model (Positive and Negative items):** χ²=732.93; CFI=0.91; TLI=0.90; RMSEA=0.05; WRMR=1.40  **EQ-15 - Guan et al. - 1 Factor Model:** χ²=422.11 CFI=0.94; TLI=0.93; RMSEA=0.08; WRMR=1.44  Convergent:  **EQ-40 x IRI-28 SS:** r= -0.26 to 0.48  **EQ-40 x Autism-Spectrum Quotient Test SS (related construct):** r= -0.45 to 0.24  **EQ-40 x Toronto Alexithymia Scale SS (related construct):** r= -0.36 to -0.29  **EQ-15 x IRI-28 SS:** r= -0.24 to 0.38  **EQ-15 x Autism-Spectrum Quotient Test SS (related construct):** r= -0.42 to 0.32  **EQ-15 x Toronto Alexithymia Scale SS (related construct):** r= -0.26 to -0.16  Known groups (Gender):  **EQ-40 - women>men:** p=0.05, cohen’s d=0.24  **EQ-15 - women=men:** p=0.84, cohen’s d=0.02 | | |
| Interperso-nal Reactivity Index (IRI) | Fernández et al. (2011)  IRI-28 | To adapt the instrument cross-culturally and to evaluate the psychometric properties of the IRI in the Chilean population | Cronbach’s alphas:  **SS-Fantasy:** α=0.76  **SS-Empathic Concern:** α=0.73  **SS-Perspective Taking:** α=0.73  **SS-Personal Distress:** α=0.70  T.R. (8w) n=82:  **SS-Fantasy:** p=0.76 to 0.82  **SS-Empathic Concern:** p=0.81 to 0.89  **SS-Perspective Taking:** p=0.67  **SS-Personal Distress:** p=0.78 to 0.81 | CFA:  **Davis Original 4 Factors Model (Empathic Concern, Fantasy, Personal Distress, Perspective Taking):** x²=781,74, p < 0.001; RMSEA=0.054; CFI=0.813; SRMR=0.070  **Cliffordson 2º order 4 Factors Model (Empathic Concern, Fantasy, Personal Distress, Perspective Taking):** x²=810,34, p < 0.001; RMSEA=0.056; CFI=0.802; SRMR=0.075  Convergent:  **IRI x Rosenberg’s Self-Esteem Scale (related construct):** r= -0.28 to 0.16  **IRI x Spielberger’s Trait Anxiety Inventory (related construct):** r= -0.12 to 0.57  **IRI x Buss and Perry Aggression Questionnaire (related construct):** r= -0.31 to 0.30  **IRI x Social Avoidance and Distress Scale (related construct):** r= -0.30 to 0.22  **IRI x Positive Affect (related construct):** r= -0.26 to 0.26  **IRI x Negative Affect (related construct):** r= -0.16 to 0.35  Known groups (Gender):  **SS-Fantasy - women>men:** p<0.001  **SS-Empathic Concern - women>men:** p<0.001  **SS-Personal Distress - women>men:** p<0.001  **SS-Perspective Taking - women=men:** NS | | |
|  | Sampaio et al. (2011)  IRI-26 | To translate, adapt and investigate the psychometric properties of IRI for the Brazilian population | Cronbach’s alphas:  *Sample 1:*  **IRI-26:** α=0.86  **SS-Fantasy:** α=0.81  **SS-Empathic Concern:** α=0.75  **SS-Perspective Taking:** α=0.68  **SS-Personal Distress:** α=0.76  *Sample 2:*  **IRI-26:** α=0.85  **SS-Fantasy:** α=0.81  **SS-Empathic Concern:** α=0.71  **SS-Perspective Taking:** α=0.71  **SS-Personal Distress:** α=0.76 | EFA: Sample 1:  KMO=0.828; Bartlett test of sphericity (p<0.001)  4 Factors Model (Fantasy, Empathic Concern, Perspective Taking, Personal Distress) = 44% of variance (Items 11 and 18 excluded)  CFA: Sample 2:  **1 Factor Model (26 items):** x²/gl=1,47; RMR=0,07; GFI=0,90; AGFI=0,86; CFI=0,94; RMSEA=0,04; ECVI=2,74; CAIC=1048,14  **2 Factors Model (Empathic Concern + Personal Distress and Fantasy + Perspective Taking - 26 items):** x²/gl=1,09; RMR=0,07; GFI=0,93; AGFI=0,89; CFI=0,98; RMSEA=0,02; ECVI=2,34; CAIC=1309,46  **Davis Original 4 Factors Model (Empathic Concern, Fantasy, Personal Distress, Perspective Taking):** x²/gl=0,89; RMR=0,05; GFI=0,94; AGFI=0,91; CFI=0,99; RMSEA=0,01; ECVI=1,97; CAIC=999,56  Known groups (Gender):  **IRI total - women>men:** p<0.001  **SS-Fantasy - women>men:** p<0.001  **SS-Empathic Concern - women>men:** p<0.001  **SS-Personal Distress - women>men:** p<0.001  **SS-Perspective Taking - women>men:** p=0.003 | | |
|  | Gilet et al. (2013)  IRI-28 | To translate, adapt and investigate the psychometric properties of IRI for the French population - modified version (7-point likert scale score) | Cronbach’s alphas:  **SS-Fantasy:** α=0.81  **SS-Empathic Concern:** α=0.70  **SS-Perspective Taking:** α=0.71  **SS-Personal Distress:** α=0.78  T.R. (12w):  **SS-Fantasy:** ICC=0.86  **SS-Empathic Concern:** ICC=0.71  **SS-Perspective Taking:** ICC=0.77  **SS-Personal Distress:** ICC=0.85 | CFA:  **1 Factor Model:** χ²=1897.21; CFI=0.33; RMSEA=0.12; SRMR=0.14  **2 Factors Model (Cognitive Empathy: Fantasy + Perspective Taking and Affective Empathy: Empathic Concern + Personal Distress):**  χ²=1734.45; CFI=0.40; RMSEA=0.11; SRMR=0.14; Factorial loadings > 0.30  **Davis Original 4 Factors Model (Empathic Concern, Fantasy, Personal Distress, Perspective Taking):** χ²=789.0; CFI=0.81; RMSEA=0.06; SRMR=0.07  Convergent:  **IRI-SS x EQ-60:** r= -0.26 to 0.35  Known groups:  *Age:*  **SS-Fantasy - young adults>older adults:** p<0.001, n²=0.14  **SS-Personal Distress - young adults>older adults:** p<0.04, n²=0.01  *Gender:*  **SS-Fantasy: women>men:** p=0.03, n²=0.01  **SS-Empathic Concern: women>men:** p<0.001, n²=0.06 | | |
|  | Koller & Lamm (2014)  IRI-16 | To examine the psychometric properties of the German version of IRI-16 using item response theory | Cronbach’s alphas:  **SS-Fantasy:** α=0.73  **SS-Empathic Concern:** α=0.61  **SS-Perspective Taking:** α=0.71  **SS-Personal Distress:** α=0.64 | Items - equal difficult/ not distributed across the latent dimensions  **Range infit =** 0.65 to 0.96  **Range outfit =** 0.65 to 0.99  **SS Fantasy, Empathic Concern, Perspective Taking and Personal Distress:** tendency of social desirability  CFA: **3 Factors Model (Empathic Concern, Fantasy Perspective Taking):** χ²=89.84; CFI=0.99; RMSEA=0.02; SRMR=0.06  **Davis Original 4 Factors Model (Empathic Concern, Fantasy, Personal Distress, Perspective Taking):** χ²=181.23; CFI=0.99; RMSEA=0.03; SRMR=0.05  **5 Factors Model (Empathic Concern, Fantasy-feel like, Fantasy-imagine, Perspective Taking-both sides, Perspective taking-oneself):** χ²=52.17; CFI=0.99; RMSEA=0.01; SRMR=0.04  Measure invariance:  Age  SS Empathic Concern: LR-value= 15.34; p=0.17  SS Perspective Taking: LR-value= 45.63; p<0.001  SS Fantasy: LR-value= 41.90; p<0.001  SS Personal Distress: LR-value= 48.66; p<0.001  Gender  SS Empathic Concern: LR-value= 16.82; p=0.33  SS Perspective Taking: LR-value= 24.73; p=0.06  SS Fantasy: LR-value= 18.95; p=0.22  SS Personal Distress: LR-value= 47.70; p<0.001 | | |
|  | Braun et al. (2015)  IRI-28 | To examine the psychometric properties of the French version of IRI using two large Belgian samples | Cronbach’s alphas:  *Sample 1:*  **Davis Original 4 Factors Model (IRI-28):**  **SS-Fantasy:** α=0.76  **SS-Empathic Concern:** α=0.75  **SS-Perspective Taking:** α=0.65  **SS-Personal Distress:** α=0.71  *Sample 2:*  **IRI-15:**  **SS-Fantasy:** α=0.76  **SS-Empathic Concern:** α=0.60  **SS-Perspective Taking:** α=0.62  **SS-Personal Distress:** α=0.70 | EFA: Sample 1:  **IRI-15** - **4 Factors Model** (**Empathic Concern, Fantasy, Personal Distress, Perspective Taking**) = only 15 items with factorial load> 0.40, % of variance = not informed  A fifth factor was identified and disregarded for not having a conceptual hypothesis  CFA:  *Sample 1:*  **Davis Original 4 Factors Model (Empathic Concern, Fantasy, Personal Distress, Perspective Taking):** χ²=1453.25; CFI=0.81; RMSEA=0.05; SRMR=0.06  **IRI-15 - 4 Factors Model (Personal Distress, Empathic Concern, Perspective Taking, Fantasy):** χ²=196.01; CFI=0.92; RMSEA=0.05; SRMR=0.05  *Sample 2:*  **IRI-15 - 4 Factors Model (Personal Distress, Empathic Concern, Perspective Taking, Fantasy):** χ²=192.28; CFI=0.92; RMSEA=0.04; SRMR=0.05  Convergent: Sample 1:  **IRI x Marlowe-Crowne Social Desirability Scale (related construct):** r= -0.24 to 0.37  Measure invariance (Gender):  **Model 1 - baseline (configural invariance):** x²=262,54, df=168  **Model 1 vs Model 2 (equal loadings):** x²=278.04, df=179, Δχ 2=15.41, Δ df=11, p>0.01  **Model 2 vs Model 3 (equal loadings + thresholds):** x²=338.61, df=190, Δχ 2=93.03, Δ df=11, p<0.001  Known groups (Gender): Sample 1:  **SS-Fantasy - women>men:** p<0.001  **SS-Empathic Concern - women>men:** p<0.001  **SS-Personal Distress - women>men:** p<0.001  **SS-Perspective Taking - women=men:** NS | | |
|  | Formiga et al. (2015)  IRI-26 | To assess, based on an analysis of the structural model, three or four fathers are the most appropriate to measure empathy using IRI-26 | Cronbach’s alphas:  α=0.70 to 0.84 | EFA:  **IRI-21 - Ribeiro et al. - 3 Factors Model (Empathic Concern, Perspective Taking, Personal Distress):** χ²/gl=1.92; GFI=0.93; AGFI=0.91; CFI=0.92; RMR=0.07; RMSEA=0.02; CAIC=1208.51; ECVI=0.90  **Sampaio et al. - Original Model (4 factors - Empathic Concern, Fantasy, Personal Distress, Perspective Taking):** χ²/gl=1.04; GFI=0.98; AGFI=0.96; CFI=1.00; RMR=0.03; RMSEA=0.01; CAIC=915.9; ECVI=0.76 | | |
|  | Chrysikou & Thompson (2016)  IRI-28 | To assess the validity of the alternative IRI model with a cognitive and an affective factor | ---- | CFA:  **2 Factors Model (Cognitive: Perspective Taking + Fantasy; Affective: Empathy Concern + Personal Distress):** CFI=0.57; TLI=0.53; RMSEA=0.18  **Pulos et al. - 3 Factors Model (Empathic Concern, Perspective Taking, Fantasy):** CFI=0.95; TLI=0.94; RMSEA=0.11  **Davis Original 4 Factors Model (Empathic Concern, Fantasy, Personal Distress, Perspective Taking):** CFI=0.95; TLI=0.95; RMSEA=0.11  Known groups (Gender):  **SS-Perspective Taking - women>men:** p=0.006, cohen’s d=-0.28  **SS-Empathic Concern - women>men:** p<0.001, cohen’s d=-0.57  **SS-Fantasy - women>men:** p<0.001, cohen’s d=-0.40  **SS-Personal Distress - women>men:** p=0.003, cohen’s d=-0.30 | | |
|  | Ingoglia et al. (2016)  IRI-28 | To develop an abbreviated version of IRI (16 items) and present its psychometric properties | Cronbach’s alphas:  *Sample 1:*  **IRI-28:**  **SS-Fantasy:** α=0.78  **SS-Empathic Concern:** α=0.73  **SS-Perspective Taking:** α=0.71  **SS-Personal Distress:** α=0.76  **IRI-16:**  **SS-Fantasy:** α=0.79  **SS-Empathic Concern:** α=0.69  **SS-Perspective Taking:** α=0.65  **SS-Personal Distress:** α=0.71  *Sample 2*:  **IRI-16:**  **SS-Fantasy:** α=0.82  **SS-Empathic Concern:** α=0.68  **SS-Perspective Taking:** α=0.69  **SS-Personal Distress:** α=0.71  *Sample 3:*  **IRI-16:**  **SS-Fantasy:** α=0.79  **SS-Empathic Concern:** α=0.68  **SS-Perspective Taking:** α=0.68  **SS-Personal Distress:** α=0.72 | Kurtosis= -0.70 to 0.86/ Skewness= -1.10 to 0.79  EFA: Sample 1:  **IRI-16 - 4 Factors Model (Personal Distress, Empathic Concern, Perspective Taking, Fantasy)** = 40.66% of variance, Factor loadings > 0.40  CFA:  *Sample 2:*  **IRI-16 - 4 Factors Model (Personal Distress, Empathic Concern, Perspective Taking, Fantasy):** χ²=344.89; CFI=0.93; RMSEA=0.05  *Sample 3:*  **IRI-16 - 4 Factors Model (Personal distress, Empathic concern, Perspective Taking, Fantasy):** χ²=281.60; CFI=0.992; RMSEA=0.05  Convergent: Sample 3:  **IRI-16 SS x Self-other Differentiation Scale (related construct):** r= -0.44 to -0.13  **IRI-16 SS x Scale for the Measurement of Emotional Fragility (related construct):** r=0.15 to 0.62  **IRI-16 SS x Bem Sex Role Inventory (related construct):** r= -0.46 to 0.83  Measure invariance:  *Age:*  **Model 1 - baseline (configural invariance):** x²=456.31, p<0.001, df=196, CFI=0.93, RMSEA=0.04  **Model 2 (full metric invariance):** x²=493.40, p<0.001, df=212, CFI=0.93, RMSEA=0.04, Δχ 2=31.36, p<0.05, Δ df=16  **Model 3 (factor variance-covariance invariance):** x²=497.98, p<0.001, df=218, CFI=0.93, RMSEA=0.04, Δχ 2=34.95, p<0.05, Δ df=22  **Model 4 (intercept invariance):** x²=533.80, p<0.001, df=234, CFI=0.93, RMSEA=0.04, Δχ 2=72.79, p<0.01, Δ df=38  **Model 5 (residual variance invariance):** x²=549.54, p<0.001, df=250, CFI=0.93, RMSEA=0.04, Δχ 2=81.95, p<0.01, Δ df=54  *Gender:*  **Model 1 - baseline (configural invariance):** x²=456.75, p<0.001, df=196, CFI=0.92, RMSEA=0.04  **Model 2 (full metric invariance):** x²=490.66, p<0.001, df=212, CFI=0.92, RMSEA=0.04, Δχ 2=30.36, p<0.05, Δ df=16  **Model 3 (factor variance-covariance invariance):** x²=502.68, p<0.001, df=218, CFI=0.92, RMSEA=0.04, Δχ 2=38.62, p>0.05, Δ df=22  **Model 4 (intercept invariance):** x²=696.43, p<0.001, df=234, CFI=0.92, RMSEA=0.04, Δχ 2=250.00, p<0.001, Δ df=38  **Model 5 (residual variance invariance):** x²=779.90, p<0.001, df=250, CFI=0.91, RMSEA=0.05, Δχ 2=279.24, p<0.001, Δ df=54  **Model 6 (partial residual variance invariance):** x²=752.74, p<0.001, df=249, CFI=0.92, RMSEA=0.05, Δχ 2=265.39, p<0.001, Δ df=53  Known groups (Gender): Sample 2:  *IRI-16:*  **SS-Perspective Taking - women>men:** cohen’s d=-0.24  **SS-Empathic Concern - women>men:** cohen’s d=-0.56  **SS-Fantasy - women>men:** cohen’s d=-0.53  **SS-Personal Distress - women>men:** cohen’s d=-0.59 | | |
|  | Budagovskaia et al. (2017)  IRI-28 | To adapt and evaluate the psychometric quality of the Russian version of IRI | Cronbach's alphas:  **SS-Fantasy:** α=0.79  **SS-Empathic Concern:** α=0.67  **SS-Perspective Taking:** α=0.60  **SS-Personal Distress:** α=0.70 | EFA:  *Sample1:*  **8 Factors - % of variance =** not informed, Factorial loadings > 0.36  **Qualitative analysis of 8 factors - grouped into 4 factors** = Davis Original 4 Factors Model (Empathic Concern, Fantasy, Personal Distress and Perspective Taking)  *Sample 2:*  **4 predefined factors - maximum factor load***:*  **SS-Empathic Concern**=0.64  **SS-Fantasy**=0.60  **SS-Perspective Taking**=0.63  **SS-Personal Distress**=0.72  Convergent:  **IRI SS x Questionnaire Measure of Emotional Empathy:** ρ=0.16 to 0.61  **IRI SS x Boiko Questionnaire Scales:** ρ=0.15 to 0.52  **IRI SS-Empathic Concern x Emotional Intelligence Questionnaire (related construct):** NS  **IRI SS-Fantasy x Emotional Intelligence Questionnaire (related construct):** NS  **IRI SS-Personal Distress x Emotional Intelligence Questionnaire (related construct):**  ρ= -0.53 to -0.15  **IRI SS-Perspective Taking x Emotional Intelligence Questionnaire (related construct):** ρ=0.10 to 0.23  Known groups (Gender):  **SS-Perspective Taking - women=men:** NS  **SS-Empathic Concern - women>men:** p=0.01  **SS-Fantasy - women>men:** p<0.001  **SS-Personal Distress - women>men:** p<0.001 | | |
|  | Molina et al. (2017)  IRI-28 | To examine the dimensional structure and measurement invariance of the IRI across gender | Cronbach's alphas:  **SS-Fantasy:** α=0.79  **SS-Empathic Concern:** α=0.72  **SS-Perspective Taking:** α=0.74  **SS-Personal Distress:** α=0.72 | CFA:  *College students:*  **Davis Original 4 Factors Model (Empathic Concern, Fantasy, Personal Distress, Perspective Taking):** χ²=2957.0; CFI=0.82; TLI=0.80; RMSEA=0.05; SRMR=0.06  **Hawk et al. - 4 Factors 2º order Model (4 factors - Empathic Concern, Fantasy, Personal Distress, Perspective Taking)**: χ²=3043.1; CFI=0.81; TLI=0.80; RMSEA=0.05; SRMR=0.06  **ESEM Original version - 4 Factors Model (invariance of this model across gender in college sample):** χ²=2075.1; CFI=0.89; TLI=0.85; RMSEA=0.05; SRMR=0.03  *Adults:*  **Davis Original 4 Factors Model (Empathic Concern, Fantasy, Personal Distress, Perspective Taking):** χ²=2444.7; CFI=0.72; TLI=0.70; RMSEA=0.06; SRMR=0.08  **Hawk et al. - 4 Factors 2º order Model (Empathic Concern, Fantasy, Personal Distress, Perspective Taking)**: χ²=2491.3; CFI=0.72; TLI=0.69; RMSEA=0.06; SRMR=0.08  **ESEM Original version - 4 Factors Model (invariance of this model across gender in college sample):** χ²=1386.7; CFI=0.88; TLI=0.83; RMSEA=0.05; SRMR=0.03  Measure invariance (*Gender):*  **Model 1 (configural invariance):** x²=2,371.8, df=544, CFI=0.88, TLI=0.84, RMSEA=0.05  **Model 2 (weak invariance):** x²=2,532.2, df=640, CFI=0.88, TLI=0.86, RMSEA=0.04  **Model 3 (strong invariance):** x²=2,679.2, df=664, CFI=0.87, TLI=0.86, RMSEA=0.04  Known groups (Gender):  **SS-Perspective Taking - women=men:** NS  **SS-Empathic Concern - women>men:** p<0.001  **SS-Fantasy - women>men:** p<0.001  **SS-Personal Distress - women>men:** p<0.001 | | |
| Questio-nnaire of Cognitive and Affective Empathy (QCAE) | Reniers et al. (2011) | To propose a new instrument for assessing empathy, the QCAE and assess its factorial structure and other psychometric qualities | Cronbach's alphas: Sample 1:  **SS-Perspective Taking:** α=0.85  **SS-Emotion Contagion:** α=0.72  **SS-Online Simulation:** α=0.83  **SS-Peripheral Responsivity:** α=0.65  **SS-Proximal Responsivity:** α=0.70 | EFA: Sample 1:  KMO=0.89; Bartlett test of sphericity (p<0.001)  **5 Factors Model (Perspective Taking, Emotion Contagion, Online Simulation, Peripheral Responsivity, Proximal Responsivity)** = 41.90% of variance, Factor loadings > 0.44  CFA: Sample 2:  **QCAE - 5 Factors Model - 1º order (Perspective Taking, Emotion Contagion, Online Simulation, Peripheral Responsivity, Proximal Responsivity):** χ²=193.90(p<0.001); RMSEA=0.07; CFI=0.95; TLI=0.93; SRMR=0.03; AIC=273.90  **QCAE - 5 Factors Model - 2º order (Cognitive Empathy: Perspective Taking + Online Simulation and Affective Empaty: Emotion Contagion + Peripheral Responsivity + Proximal Responsivity):** χ²=244.31(p<0.001); RMSEA=0.08; CFI=0.92; TLI=0.91; SRMR=0.04; AIC=314.31  Convergent:  **QCAE SS x Basic Empathy Scale SS-Cognitive Empathy:** r=0.62  **QCAE SS x Basic Empathy Scale SS-Affective Empathy:** r=0.76  **QCAE Total and SS x Trait Empathic Anger:** r=0.22 to 0.41  **QCAE Total and SS x Barratt Impulsiveness Scale Total and SS (related construct):** r= -0.29 to -0.09  **QCAE Total and SS x Impulsivity Inventory (related construct):** r= -0.40 to -0.09  **QCAE Total and SS x Life History of Aggression Questionnaire (related construct):** r= -0.19 to -0.10  **QCAE Total and SS x Short Expagg (aggressive) Questionnaire- SS-Instrumental (related construct):** r= - 0.23 to -0.10  **QCAE Total and SS x Levenson Psychopathy Scale (related construct):** r= -0.42 to -0.12  **QCAE Total and SS x Machiavellianism Test (related construct):** r= -0.32 to - 0.10  Known groups (Gender):  **SS-Cognitive scale - women>men:** p=0.001, cohen’s d=0.41  **SS-Affective scale - women>men:** p=0.001, cohen’s d=0.83 | | |
|  | Girolamo et al. (2017) | To examine the cross-cultural adaptability of the QCAE, of an Italian version in two samples and with two different formats of administration, via paper-and-pencil and via an online format | Cronbach's alphas:  *Paper-and-pencil dataset:*  **Total:** α=0.87  **SS:** α=0.58 to 0.89  *Online dataset:*  **Total:** α=0.86  **SS:** α=0.69 to 0.84  *Combined dataset:*  **Total:** α=0.87  **SS:** α=0.63 to 0.87 | CFA:  *Paper-and-pencil dataset:*  **QCAE - 1 Factor Model:** χ²=1400.55; CFI=0.78; NNFI=0.75; AIC=1460.55; RMSEA=0.19; SRMR=0.13  **QCAE - Reniers et al. Original 5 Factors Model - 1º order (Perspective Taking, Emotion Contagion, Online Simulation, Peripheral Responsivity, Proximal Responsivity):** χ²=257.67; CFI=0.96; NNFI=0.94; AIC=337.67; RMSEA=0.07; SRMR=0.07  **QCAE - Reniers et al. Original 5 Factors Model - 2º order (Cognitive Empathy: Perspective Taking + Online Simulation and Affective Empaty: Emotion Contagion + Peripheral Responsivity + Proximal Responsivity):** χ²=292.85; CFI=0.95; NNFI=0.94; AIC=362.26; RMSEA=0.08; SRMR=0.08  *Online dataset:*  **QCAE - 1 Factor Model:** χ²=1400.55; CFI=0.78; NNFI=0.75; AIC=1460.55; RMSEA=0.19; SRMR=0.13  **QCAE - Reniers et al. Original 5 Factors Model - 1º order (Perspective Taking, Emotion Contagion, Online Simulation, Peripheral Responsivity, Proximal Responsivity):** χ²=257.67; CFI=0.96; NNFI=0.94; AIC=337.67; RMSEA=0.07; SRMR=0.07  **QCAE - Reniers et al. Original 5 Factors Model - 2º order (Cognitive Empathy: Perspective Taking + Online Simulation and Affective Empaty: Emotion Contagion + Peripheral Responsivity + Proximal Responsivity):** χ²=292.85; CFI=0.95; NNFI=0.94; AIC=362.26; RMSEA=0.08; SRMR=0.08  Convergent:  *Paper-and-pencil dataset:*  **QCAE x Interpersonal Competence Questionnaire SS (related construct):** r=0.24 to 0.56  **QCAE SS x Interpersonal Competence Questionnaire SS (related construct):** r=-0.12 to 0.57  **QCAE x NEO-FFI factors (related construct):** r= -0.05 to 0.31  **QCAE SS x NEO-FFI factors (related construct):** r= -0.27 to 0.41  **QCAE x Psychological General Well-Being Index SS (related construct):** r= -0.02 to 0.10  **QCAE SS x Psychological General Well-Being Index SS (related construct):** r= -0.23 to 0.20  **QCAE x Difficulties in Emotion Regulation Scale SS (related construct):** r= -0.37 to 0.04  **QCAE SS x Difficulties in Emotion Regulation Scale SS (related construct):** r= -0.37 to 0.25  *Online dataset:*  **QCAE x Toronto Alexithymia Scale SS (related construct):** r= -0.43 to -0.04  **QCAE SS x Toronto Alexithymia Scale SS (related construct):** r= -0.43 to 0.15  **QCAE x Reading the Mind in the Eyes (related construct):** r=0.16  Measure invariance (Paper-and-pencil dataset x online dataset):  **Configural Invariance:** χ²=526.7; CFI=0.95; NNFI=0.93; AIC=686.7; RMSEA=0.08; SRMR=0.13  **Metric Invariance:** χ²=538.1; CFI=0.95; NNFI=0.93; AIC=678.1; RMSEA=0.08; SRMR=0.08  **Scalar Invariance:** χ²=538.1; CFI=0.95; NNFI=0.94; AIC=718.1; RMSEA=0.19; SRMR=0.08  **Invariant Unique Variance:** χ²=565.7; CFI=0.95; NNFI=0.94; AIC=718.1; RMSEA=0.07; SRMR=0.08  Comparison between QCAE Scores from Paper-and-Pencil and Online Administrations:  **QCAE total and SS-Perspective Taking, Proximal Responsivity, Cognitive Empathy, Affective Empathy:**  Paper-and-pencil dataset<Online dataset: p<0.01  **SS-Online Simulation, Emotion Contagion, Peripheral Responsivity**  Paper-and-pencil dataset=Online dataset: NS | | |
|  | Myszkowski et al. (2017) | To build a French translation and adaptation of the QCAE, and to further investigate its factor structure | Cronbach's alphas:  **SS-Perspective Taking:** α=0.89  **SS-Emotion Contagion:** α=0.74  **SS-Online Simulation:** α=0.84  **SS-Peripheral Responsivity:** α=0.62  **SS-Proximal Responsivity:** α=0.71 | CFA:  **QCAE - Reniers et al. Original 5 Factors Model (Perspective Taking, Emotion Contagion, Online Simulation, Peripheral Responsivity, Proximal Responsivity):** χ²=277.33; χ²/df=3.46; CFI=0.93; SRMR=0.05; RMSEA=0.07; AIC=9706.0  **QCAE - 5 Factors Model - 2º order (Cognitive Empathy: Perspective Taking + Online Simulation and Affective Empathy: Emotion Contagion + Peripheral Responsivity + Proximal Responsivity):** χ²=305.24; χ²/df=3.63; CFI=0.93; SRMR=0.06; RMSEA=0.07; AIC=9726.0  **QCAE - 5 Factors Model - with 1 inequality constraint (Perspective Taking, Emotion Contagion, Online Simulation, Peripheral Responsivity, Proximal Responsivity):** χ²=341.80; χ²/df=4.02; CFI=0.91; SRMR=0.07; RMSEA=0.08; AIC=9760.5  **QCAE - 5 Orthogonal Factors Model (Perspective Taking, Emotion Contagion, Online Simulation, Peripheral Responsivity, Proximal Responsivity):** χ²=920.99; χ²/df=10.23; CFI=0.73; SRMR=0.28; RMSEA=0.14; AIC=10329.7  **QCAE - 5 Factors Model - 2º order - with 1 inequality constraint (Cognitive Empathy: Perspective Taking + Online Simulation and Affective Empathy: Emotion Contagion + Peripheral Responsivity + Proximal Responsivity):** χ²=448.9; χ²/df=5.28; CFI=0.88; SRMR=0.18; RMSEA=0.10; AIC=9897.6  **QCAE - 1 Factor Model:** χ²=997.96; χ²/df=11.09; CFI=0.71; SRMR=0.10; RMSEA=0.15; AIC=10436.7  **QCAE - 2 Orthogonal Factors Model (Cognitive Empathy, Affective Empathy):** χ²=822.21; χ²/df=9.14; CFI=0.76; SRMR=0.20; RMSEA=0.14; AIC=10230.9  **QCAE - 2 Correlated Factors Model (Cognitive Empathy, Affective Empathy):** χ²=721.39; χ²/df=8.11; CFI=0.80; SRMR=0.10; RMSEA=0.13; AIC=10132.1  Measure invariance (Gender):  **Model 1 (equal factor loadings):** Δχ2=368.94, Δdf=10, p=0.30, ΔCFI=0.001, ΔRMSEA=0.002, ΔSRMR=0.003  **Model 2 (equal factor loadings and intercepts):** Δχ2=17.47, Δdf=10, p=0.06, ΔCFI=0.002, ΔRMSEA=0.001, ΔSRMR=0.002  **Model 3 (equal factor loadings, intercepts and means):** Δχ2=428.69, Δdf=5, p<0.001, ΔCFI=0.012, ΔRMSEA=0.005, ΔSRMR=0.010 | | |
|  | Queirós et al. (2018) | Cross-culturally adapt and investigate the psychometric properties of QCAE, in a Portuguese community sample | Cronbach's alphas:  **Total:** α=0.87  **SS:** α=0.62 to 0.87  Composite reliability**:**  **QCAE - 5 correlated factors**  **Total** =0.95 / **SS**=0.64 to 0.87  **QCAE - 5 factors with 2 correlated second-order factors:**  **Total**=0.95 / **SS**=0.67 to 0.91 | CFA:  **QCAE - Reniers et al. Original 5 Factors Model (Perspective Taking, Emotion Contagion, Online Simulation, Peripheral Responsivity, Proximal Responsivity):** χ²=3466.08; CFI=0.91; TLI=0.90; SRMR=0.09; RMSEA=0.09  **QCAE - 5 Factors Model - with 2 correlated 2º order (Cognitive Empathy: Perspective Taking + Online Simulation and Affective Empathy: Emotion Contagion + Peripheral Responsivity + Proximal Responsivity):** χ²=3413.28; CFI=0.90; TLI=0.90; SRMR=0.09; RMSEA=0.09  Measure invariance (Gender):  *Model 1 (5 correlated version):*  **Total sample:** x²=193.90, p<0.001, df=80, CFI=0.94, TLI=0.97, RMSEA=0.04  **Male sample:** x²=161.25, p<0.001, df=80, CFI=0.96, TLI=0.95, RMSEA=0.05  **Female sample:** x²=209.36, p<0.001, df=80, CFI=0.98, TLI=0.97, RMSEA=0.04  *Model 2 (5 factors with 2 correlated second-order factors):*  **Total sample:** x²=339.46, p<0.001, df=85, CFI=0.97, TLI=0.96, RMSEA=0.05  **Male sample:** x²=166.49, p<0.001, df=85, CFI=0.96, TLI=0.95, RMSEA=0.05  **Female sample:** x²=212.31, p<0.001, df=85, CFI=0.98, TLI=0.97, RMSEA=0.04  Known groups (Gender):  **QCAE - women>men:** p<0.001, cohen’s d=-0.65  **SS-Cognitive Empathy - women>men:** p<0.001, cohen’s d=0.31  **SS-Affective Empathy - women>men:** p<0.001, cohen’s d=0.84  **SS-Perspective Taking - women>men:** p<0.001, cohen’s d=0.30  **SS-Online Simulation - women>men:** p<0.001, cohen’s d=0.22  **SS-Emotional Contagion - women>men:** p<0.001, cohen’s d=0.58  **SS-Proximal Responsivity - women>men:** p<0.001, cohen’s d=0.63  **SS-Peripheral Responsivity - women>men:** p<0.001, cohen’s d=0.67 | | |
|  | Liang et al. (2019) | To adapt and study the psychometric properties of QCAE, in a Chinese community sample | Cronbach's alphas:  **Total:** α=0.86  **SS:** α=0.52 to 0.88  TR. (4w):  **Total:** r=0.76  **SS:** r=0.65 to 0.75 | EFA:  KMO=0.89; Bartlett test of sphericity (p=0.001)  **4 factors (Perspective Taking, Online Simulation, Peripheral Responsivity, Emotional Contagion+ Proximal Responsivity)**= 46.41% variance, Factor loadings ≥ 0.30  CFA:  **QCAE - Reniers et al. Original 5 Factors Model (Perspective Taking, Emotion Contagion, Online Simulation, Peripheral Responsivity, Proximal Responsivity):** χ²=278.16; CFI=0.93; TLI=0.91; SRMR=0.06; RMSEA=0.07  **QCAE - 5 Factors Model - 2º order (Cognitive Empathy: Perspective Taking + Online Simulation and Affective Empathy: Emotion Contagion + Peripheral Responsivity + Proximal Responsivity):** χ²=309.64; CFI=0.92; TLI=0.90; SRMR=0.07; RMSEA=0.07  **QCAE - 4 Factors Model - 1º order (Perspective Taking, Online Simulation, Emotion Contagion/Proximal Responsivity, Peripheral Responsivity):** χ²=328.64; CFI=0.92; TLI=0.89; SRMR=0.06; RMSEA=0.07  **QCAE - 4 Factors Model - 2º order (Cognitive Empathy: Perspective Taking + Online Simulation and Affective Empathy: Emotion Contagion/Proximal + Peripheral Responsivity):** χ²=329.66; CFI=0.92; TLI=0.90; SRMR=0.06; RMSEA=0.07  Convergent:  **QCAE total and SS x IRI SS:** r= -0.27 to 0.70  **QCAE x Revised Social Anhedonia (related construct):** r= -0.27 to -0.12  Measure invariance (Gender):  **Model 1 (metric invariance):** Δχ2=7,03, Δdf=9, p> 0,05, ΔCFI=0,001, ΔTLI=0,007  **Model 2 (scalar invariance):** Δχ2=20.41, Δdf=9, p <0 .05, ΔCFI= −.004, ΔTLI= −0.000)  **Model 3 (error variance invariance):** Δχ2=26.23, Δdf=13, p < .05, ΔCFI= −.004, ΔTLI=0.002  Known groups (Gender):  **QCAE - women>men:** p<0.001, cohen’s d=-0.23  **SS-Cognitive Empathy - women=men:** NS  **SS-Affective Empathy - women>men:** p<0.001  **SS-Emotional Contagion - women>men:** p<0.001, cohen’s d=-0.23  **SS-Proximal Responsivity - women>men:** p<0.001, cohen’s d=-0.21  **SS-Peripheral Responsivity - women>men:** p<0.001, cohen’s d=-0.46 | | |
| The Active-Empathic Listening Scale (AELS) | Bodie (2011) | To adapt the AELS (self and other-report) to the general context of interpersonal relationships and to evaluate its psychometric properties | Cronbach's alphas**:**  *Sample 1:*  **Total:** α=0.86  **SS-Sensing:** α=0.73  **SS-Processing:** α=0.66  **SS-Responding:** α=0.78  *Sample 2:*  **Total:** α=0.94  **SS-Sensing:** α=0.85  **SS-Processing:** α=0.77  **SS-Responding:** α=0.89 | CFA:  *Sample 1:*  **AELS-self-report- 3 Factors Model (Sensing, Processing, Responding):** X²=119,10, p < 0.001; GFI=0.95; AGFI=0.92; CFI=0.95; RMR=0.062; RMSEA=0.06; Factor loadings ≥ 0.54  *Sample 2:*  **AELS-other-report - 3 Factors Model (Sensing, Processing and Responding):** x²=96,40, p < 0.001; GFI=0.93; AGFI=0.89; CFI=0.97; RMR=0.086; RMSEA=0.07; Factor loadings ≥ 0.60  Convergent:  *Sample 1:*  **AELS-self-report x Empathic Responsiveness:** r=0.15 to 0.18  **AELS-self-report x Perspective Taking:** r=0.28 to 0.44  **AELS-self-report x Sympathetic Responsiveness:** r=0.18 to 0.40  **AELS-self-report x Interaction Involvement SS (related construct)**: r=0.13 to 0.67  **AELS-self-report x Conversational Sensitivity SS (related construct):** r=0.17 to 0.45  **AELS-self report x Talkaholic Scale (related construct):** r=0.13  *Sample 2:*  **AELS-other-report x Conversational Appropriateness (related construct):** r=0.53 to 0.65  **AELS-other-report x Conversational Effectiveness (related construct):** r=0.69 to 0.75  **AELS-other-report x Nonverbal Immediacy (related construct):** r=0.15 to 0.19  Measure invariance (Self-report x Other-report): Sample 2:  **Model 1 - baseline (unconstrained):** x²=215,54, p < 0.001; GFI=0.94; AGFI=0.91; CFI=0.96; RMR=0.075; RMSEA=0.05  **Model 2 - the first-order factor weights to be equivalent (measurement weights):**  Δχ 2 (8, N=217)=20.42, p>0.01  **Model 3 - the second-order factor weights to be equivalent (structural weights):**  Δχ 2 (2, N=217)=1.37, p=0.50  Known groups: Sample 2:  **Good listener > Bad listener:** p<0.001, correct rating rate=88,5% | | |
|  | Gearhart & Bodie (2011) | To provide further validity evidence for a AELS-self-report, investigating the degree to which AELS is empirically related to various general social skills that reflect interaction competencies such as emotional sensitivity | ---- | CFA:  **AELS - 3 Factors Model (Sensing, Processing, Responding)**: CFI=0.96; RMSEA=0.05  Convergent:  **AELS Total x Social Skills Inventory (related construct):** r= -0.16 to 0.49  **SS-Sensing x Social Skills Inventory (related construct):** r= 0.12 to 0.48  **SS-Processeing x Social Skills Inventory (related construct)**: r= 0.14 to 0.33  **SS-Responding x Social Skills Inventory (related construct):** r= 0.16 to 0.44 | | |
| The Toronto Empathy Questio-nnaire (TEQ) | Spreng et al. (2009) | To develop a TEQ and assess its psychometric properties | Cronbach's alphas:  **Sample 1:** α=0.85  **Sample 2:** α=0.85  **Sample 3:** α=0.87 | EFA:  *Sample 1:*  TEQ-16 - 1 Factor Model - factor loadings ranged from 0.41 a 0.65  *Sample 2:*  TEQ-16 - 1 Factor Model - factor loadings ranged from 0.37 a 0.71  Convergent:  *Sample 1:*  **TEQ-16 x IRI SS:** r=0.35 to 0.74  **TEQ-16 x Autism Quotient (related construct):** r= -0.30  *Sample 2:*  **TEQ-16 x IRI SS:** r=0.29 to 0.74  **TEQ-16 x Reading the Mind in the Eyes (related construct):** r= 0.35  **TEQ-16 x Interpersonal Perception Task–15 (related construct):** r=0.23  *Sample 3:*  **TEQ-16 x EQ:** r= 0.80  **TEQ-16 x Autism Quotient (related construct):** r= -0.33  Known groups (Gender):  *Sample 1:*  **TEQ-16 - women=men:** NS  *Sample 2:*  **TEQ-16 - women>men:** cohen’s d=0.73  *Sample 3:*  **TEQ-16 - women>men:** cohen’s d=0.63 | | |
|  | Totan et al. (2012) | To translate, adapt and investigate the psychometric properties of TEQ for the Turkish population | Cronbach's alphas:  α=0.79  T.R. (3w) n=77:  **Total:** r=0.73 | EFA:  **TEQ-16 - 1 Factor Model:**  KMO=0.85; Bartlett test of sphericity (χ²=1519.05, p<0.001)  24.58% of variance, Factor loadings > 0.40 (except for items 1, 6 and 9)  **TEQ-13 - 1 Factor Model (excluding items 1, 6 and 9):**  KMO=0.85; Bartlett test of sphericity (χ²=1350.23, p<0.001)  29.17% of variance, Factor loadings > 0.56  CFA:  **TEQ-13 - 1 Factor Model:** χ²=265.34; GFI=0.93; NFI=0.91 RFI=0.89; CFI=0.93; IFI= 0.93; RMR=0.05; RMSEA=0.07  **TEQ-13 - 1 Factor Model (with error covariances were associated):** χ²=234.67; GFI=0.94; NFI=0.91 RFI=0.90; CFI=0.94; IFI= 0.94; RMR=0.05; RMSEA=0.07  **TEQ-16 - 1 Factor Model:** χ²=405.92; GFI=0.92; NFI=0.88; RFI=0.86; CFI=0.91; RMR=0.06; RMSEA=0.07  Convergent:  **TEQ-16 x Empathic Tendency Scale:** r=0.35  **TEQ-16 x Basic Empathy Scale:** r=0.68  **TEQ-16 SS-Cognitive x Basic Empathy Scale:** r=0.47  **TEQ-16 SS-Affective x Basic Empathy Scale:** r=0.59  Known groups (Gender):  **TEQ-16 - women>men:** d=0.57 | | |
| Empathy Assessment Index (EAI) | Gerdes et al. (2011)  EAI-54 | To present the development of EAI and to investigate the psychometric properties of this instrument through an initial pilot study | Cronbach's alfas:  *Complete version:*  **SS-Affective Response:** α=0.82  **SS-Empathic Attitudes:** α=0.79  **SS-Perspective Taking:** α= 0.39  **SS-Emotion Regulation:** α= 0.70  **SS-Self-Awareness:** α= 0.30  *With item removed:*  **SS-Affective Response:** α=0.83  **SS-Empathic Attitudes:** α=0.81  **SS-Perspective Taking:** α= 0.81  **SS-Emotion Regulation:** α= 0.70  **SS-Self-Awareness:** α= 0.30  T.R. (1w):  r=0.59 to 0.85 | EFA:  **6 Factors Model (Empathetic Attitudes, Affective Response - happy, Perspective Taking, Affective Response - sad, Perspective Taking/Affective Response and Emotion Regulation)** = 43.19% of variance, Factor loadings > 0.40  Convergent:  **EAI SS-Affective Response x IRI SS-Empathic Concern:** r= 0.48  **EAI SS-Empathetic Attitudes x IRI SS-Empathic Concern:** r= 0.57  **EAI SS-Perspective Taking x IRI SS-Perspective Taking:** r= 0.75 | | |
|  | Lietz et al. (2011)  EAI-50 | To examine the psychometric properties of a revised version of the EAI. | Cronbach's alphas:  **EAI-48 – 5 Factors Model:**  **SS-Affective Response:** α=0.84  **SS-Emotion Regulation:** α=0.72  **SS-Empathic Attitude:** α=0.79  **SS-Perspective Taking:** α=0.82  **SS-Self-other Awareness:** α=0.70  T.R. (1w):  r=0.67 to 0.79 | CFA: (1st half of the sample):  **EAI-40 - 5 Factors Model (Affective Response, Emotion Regulation, Empathic Attitude, Perspective Taking, Self-other Awareness):** x²=2943.00(p<0.01); CFI=0.77; WRMR=2.19; RMSEA=0.09  **EAI-24 - 5 Factors Model (Affective Response, Emotion Regulation, Empathic Attitude, Perspective Taking, Self-other Awareness):** x²=694.11(p<0.01); CFI=0.92; WRMR=1.33; RMSEA=0.07  **EAI-24 - 5 Factors Model (Affective Response, Emotion Regulation, Empathic Attitude, Perspective Taking, Self-other Awareness) - with eight error covariance added**: x²=400.14(P<0.01); CFI=0.96; WRMR=0.94; RMSEA=0.04  **EAI-17 - 5 Factors Model (Affective Response, Emotion Regulation, Empathic Attitude, Perspective Taking, Self-other Awareness):** x²=213.70(p<0.01); CFI=0.97; WRMR=0.88; RMSEA=0.05  **EAI-17 - 5 Factors Model (Affective Response, Emotion Regulation, Empathic Attitude, Perspective Taking, Self-other Awareness) - with correlated error:** x²=185.16(p<0.01); CFI=0.98; WRMR=0.80; RMSEA=0.04  CFA: (2nd half of the sample):  **EAI-24 - 5 Factors Model** **(Affective Response, Emotion Regulation, Empathic Attitude, Perspective Taking, Self-other Awareness):** x²=694.11(p<0.01); CFI=0.87; WRMR=1.55; RMSEA=0.08  **EAI-17 - 5 Factors Model** **(Affective Response, Emotion Regulation, Empathic Attitude, Perspective Taking, Self-other Awareness)**: x²=301.68(p<0.01); CFI=0.92; WRMR=1.33; RMSEA=0.07  **EAI-17 - 5 Factors Model - with eight error covariance (Affective Responde, Emotion Regulation, Empathic Attitude, Perspective Taking, Self-other Awareness)**: x²=185.16(p<0.01); CFI=0.95; WRMR=0.97; RMSEA=0.06  Convergent: 2nd half of the sample:  **EAI SS-Emotion regulation x Cognitive Emotion Regulation Questionnaire-short (related construct):** r=0.51  **EAI SS-Emotion regulation x Mindfulness Attention and Awareness Scale (related construct):** r= -0.27  **EAI SS-Self-other awareness x Mindfulness Attention and Awareness Scale (related construct):** r= -0.40  Known groups: 2nd half of the sample:  *Gender:*  **EAI total - women>men:** p=0.05  **SS-Self-other awareness - women>men:** p=0.06  *Race:*  **SS-Empathic attitude - African Americans and Latinos>Caucasians:** p=0.001  *College majors:*  **SS-Empathic attitude - Social workers>Criminal justice, sociology, education and nursing:** p=0.001  *Family-of-origin socioeconomic status:*  **SS-Empathic attitude - Poor or working class>Middle class, Upper-middle class and wealthy:** p=0.001 | | |
| Affective and Cognitive Measure of Empathy (ACME) | Vachon & Lynam (2016) | To develop and evaluate the psychometric properties of the ACME | Cronbach's alphas:  *Sample 1:*  **SS-Cognitive Empathy:** α=0.90  **SS-Affective Resonance:** α=0.87  **SS-Affective Dissonance:** α=0.87  *Sample 2:*  **SS-Cognitive Empathy:** α=0.91  **SS-Affective Resonance:** α=0.86  **SS-Affective Dissonance:** α=0.87  *Sample 3:*  **SS-Cognitive Empathy:** α=0.90  **SS-Affective Resonance:** α=0.85  **SS-Affective Dissonance:** α=0.86 | CFA:  *Sample 1:*  **3 Factors Model (Cognitive Empathy, Affective Resonance, Affective Dissonance):** CFI=0.95; TLI=0.95; RMSEA=0.05  *Sample 2:*  **3 Factors Model (Cognitive Empathy, Affective Resonance, Affective Dissonance):** CFI=0.97; TLI=0.97; RMSEA=0.03; Factor loadings ranged from 0.45 to 0.88  Convergent:  *Sample 1:*  **ACME SS x IRI SS-Cognitive Empathy:** r=0.32 to 0.80  **ACME SS x Basic Empathy Scale SS:** r=0.40 to 0.65  **ACME SS x Aggressive Behavior (related construct):** r= -0.57 to -0.30  **ACME SS x Externalizing Disorders (related construct):** r= -0.68 to 0.06  *Sample 3:*  **ACME SS x Prosocial Behavior (related construct):** r=0.18 to 0.37  **ACME SS x Autism Behavior (related construct):** r= -0.41 to 0.00  **ACME SS x Affective Responding (related construct):** r= -0.30 to 0.27  **ACME SS x Emotion Detection (related construct):** r= -0.12 to 0.27  Measure invariance (Gender):  **Model 1 (weak invariance):** x²=109.38, p<0.001, df=67, CFI=0.96, TLI=0.95, RMSEA=0.04  **Model 2 (strong invariance):** x²=159.96, p<0.001, df=106, CFI=0.96, TLI=0.96, RMSEA=0.04  **Model 3 (strict invariance):** x²=67.34, p<0.001, *df*=36, CFI=0.97, TLI=0.97, RMSEA=0.03 | | |
|  | Murphy et al. (2018) | To investigate the psychometric properties of the ACME | ---- | CFA:  **ACME - 3 Factors Model:** χ²=3898.76; CFI=0.90; TLI=0.89; RMSEA=0.12  **ACME - 5 Factors Model (with** t**wo method factors - positive and reverse wording + Cognitive Empathy, Affective Resonance, Affective Dissonance):** χ²=1079.82; CFI=0.98; TLI=0.98; RMSEA=0.05  ESEM:  **ACME - 3 Factors Model:** χ²=1079.82; CFI=0.98; TLI=0.98; RMSEA=0.06  **ACME - 4 Factors Model**: χ²=888.99; CFI=0.99; TLI=0.99; RMSEA=0.05  Convergent:  **ACME SS-Cognitive Empathy x IRI SS:** r= 0.33 to 0.44  **ACME SS-Affective Dissonance x IRI SS:** r= -0.24 to 0.52  **ACME SS-Affective Resonance x IRI SS:** r= 0.31 to 0.77  **ACME SS x General Personality Indices (related construct):**  **ACME SS-Cognitive Empathy**: r= -0.21 to 0.36  **ACME SS-Affective Dissonance:** r= -0.83 to 0.19  **ACME SS-Affective Resonance:** r= -0.45 to 0.51  **ACME SS x Indices of Personality Disorder Features (related construct):**  **ACME SS-Cognitive Empathy**: r= -0.45 to -0.16  **ACME SS-Affective Dissonance:** r= -0.83 to -0.19  **ACME SS-Affective Resonance:** r= -0.80 to -0.17 | | |
| Measure of Empathy and Sympathy (MES) | Wang et al. (2017) | To evaluate the psychometric properties of the MES for the Chinese population | Cronbach's alphas:  **Total:** α=0.78  **Cognitive Empathy:** α=0.77  **Affective Empathy:** α=0.80  **Sympathy:** α=0.79  T.R. (4w) n=367:  **Total:** r=0.67  **Cognitive Empathy:** r=0.64  **Affective Empathy:** r=0.65  **Sympathy:** r=0.66 | EFA:  KMO=0.82; Bartlett test of sphericity (χ²=1124.36, p<0.001)  **3 Factors Model (Cognitive, Affective, Sympathy**) = 61.83% of variance  CFA:  **MES - 3 Factors Model (Cognitive, Affective, Sympathy):** χ²=77.85; CFI=0.97; TFI=0.96; RMSEA=0.04; SRMR=0.04  Convergent:  **MES SS x IRI SS-Perspective Taking:**  **SS-Cognitive Empathy:** r=0.30  **SS-Affective Empathy:** r=0.09  **SS-Sympathy:** r=0.29  **MES SS x IRI SS-Empathic Concern:**  **SS-Cognitive Empathy:** r=0.22  **SS-Affective Empathy:** r=0.22  **SS-Sympathy:** r=0.48  **MES SS x Prosocial Tendencies Measure (related construct):**  **SS-Cognitive Empathy:** r=0.32  **SS-Affective Empathy:** r=0.26  **SS-Sympathy:** r=0.30  **MES SS x Buss-Perry Aggression Questionnaire SS-Physical Aggression (related construct):**  **SS-Cognitive Empathy:** r=-0.06 (NS)  **SS-Affective Empathy:** r=0.04 (NS)  **SS-Sympathy:** r=-0.19  Known groups (Gender):  **SS-Cognitive Empathy - women>men:** p>0.01, d=-0.24  **SS-Affective Empathy - women>men:** p>0.01, d=-0.22  **SS-Sympathy - women>men:** p>0.001, d=-0.40  Predictive:  SS sympathy had incremental predictive value in measurement of prosocial behavior, and physical aggression beyond levels that could be predicated by only SS cognitive + SS affective | | |
| Multidi-mensional  Emotional Empathy Scale (MDEES) | Alloway et al. (2016) | To evaluate the psychometric properties of the MDEES | Cronbach's alphas:  α=0.88 | EFA:  **6 Factors Model (Suffering, Crying, Feeling Others, Positive Sharing, Emotional Attention, Emotional Contagion, Animals)** = 58.93% of variance, 26 items: Factor loadings > 0.45  Discriminant:  **MDEES SS x Wechsler Abbreviated Scale of Intelligence- Vocabulary (related construct):**  **SS-Empathic Suffering:** r= -0.14  **SS-Positive Sharing:** r= -0.13 | | |
| Basic Empathy Scale (BES) | Carré et al. (2013) | To validate the BES in Adults and examine its psychometric properties. | Cronbach's alphas:  **BES - 2 Factors Model:**  **SS-Cognitive Empathy:** α=0.71  **SS-Affective Empathy:** α=0.84  **BES - 3 Factors Model:**  **SS-Cognitive Empathy:** α=0.69  **SS-Emotional Contagion:** α=0.72  **SS-Emotional Disconnection:** α=0.82  T.R. (7w):  **BES - 2 Factors Model:**  **SS-Cognitive Empathy:** r=0.61  **SS-Affective Empathy:** r=0.80  **BES - 3 Factors Model:**  **SS-Cognitive Empathy:** r=0.56  **SS-Emotional Contagion:** r=0.74  **SS-Emotional Disconnection:** r=0.70 | CFA:  **BES- 1 Factor Model - 20 items (Affective Empathy, Cognitive Empathy):** χ²=776.54; RMSEA=0.10; GFI=0.93; AGFI=0.91  **BES - 2 Factors Model - 20 items (Affective Empathy, Cognitive Empathy) - original model:** χ²=510.65; RMSEA=0.07; GFI=0.95; AGFI=0.94  **BES - 3 Factors Model - 20 items (Emotional Contagion, Cognitive Empathy, Emotional Disconnection):** χ²=460.10; RMSEA=0.07; GFI=0.96; AGFI=0.95  **BES - 3 Factors Model - 19 items (Emotional Contagion, Cognitive Empathy, Emotional Disconnection):** χ²=372.28; RMSEA=0.06; GFI=0.97; AGFI=0.96  Convergent:  **BES SS x IRI SS:** r= -0.66 to 0.50  **SS-Cognitive Empathy x Emotional State Questionnaire SS-Social context (related construct):** r=0.19  **SS-Emotional Disconnection x Emotional State Questionnaire SS-Expression (related construct):** r= -0.24  **SS-Emotional Contagion x Emotional State Questionnaire SS-Expression (related construct):** r= -0.18  **SS-Emotional Contagion x Toronto Alexithymia Scale SS-Difficulties in Identifying**  **Feelings (related construct):** r=0.19  **SS-Cognitive Empathy x Toronto Alexithymia Scale SS-Difficulties in Identifying**  **Feelings (related construct):** r= -0.18  Known groups (Gender):  **SS-Cognitive Empathy - women>men:** p=0.64, cohen’s d=0.09  **SS-Emotional Contagion - women>men**: p<0.001, cohen’s d=0.70  **SS-Emotional Disconnection - women<men:** p<0.001, cohen’s d=0.64 | | |
| Empathy Inventory (EI) | Falcone et al. (2013) | To gather additional evidence about the EI construct validity, by verifying its convergent validity |  | Convergent:  **EI SS x Multidimensional Interpersonal Reactivity Scale SS**  **SS-Perspective Taking:** r= -0.19 to 0.61  **SS-Interpersonal Flexibility:** r= -0.17 to 0.31  **SS-Altruism:** NS  **SS-Affective Sensitivity:** r= 0.16 to 0.19 | | |
| Multifaceted Empathy Test (MET) | Foell et al. (2018) | To translate, adapt and investigate the psychometric properties of MET for the English population | Cronbach's alphas:  **SS-Cognitive Empathy (40 items):** α=0.49  **SS-Cognitive Empathy (after reduction**  **19 items):** α=0.51  **SS-Emotional Empathy Positive:** α=0.93  **SS-Emotional Empathy Negative:** α=0.94 | Convergent:  **MET SS x IRI SS:** r=0.22 to 0.34  **MET SS x Triarchic Psychopathy Measure SS (related construct)**: r=-0.31 to 0.29 | | |
| The Vicarious Experience Scale (VES) | Oceja Fernández et al. (2009) | To develop and assess validity evidences to VES | Cronbach's alphas:  *Sample 1:*  **SS-Sympathy:** α=0.72  **SS-Vicarious Distress:** α=0.78  *Sample 2:*  **SS-Sympathy:** α=0.72  **SS-Vicarious Distress:** α=0.78 | EFA: Sample 1:  **4 factors (Emotional Comprehension, Perspective-Taking, Sympathy, Vicarious Distress)** = 37.39% of variance, Factor loadings > 0.40  Convergent: Sample 1:  **SS-Vicarious Distress x IRI SS-Personal Distress:** r=0.23  **SS-Vicarious Distress x IRI SS-Empathic Concern:** r=0.45  **SS-Sympathy x IRI SS-Personal Distress:** r=0.14  **SS-Sympathy x IRI SS-Empathic Concern:** r=0.30  Discriminant: Sample 1:  **SS-Vicarious Distress x IRI SS-Perspective Taking**: r=0.09  **SS-Sympathy x IRI SS-Personal Distress**: r= -0.05  Predictive:  VES Sympathy and Vicarious Distress subscales have genuine predictive power above and beyond the IRI.  **Only IRI:** Empathy: R^2^=0.192 / Personal Distress: R^2^=0.138  **IRI+VES- Sympathy and Vicarious Distress**: Empathy: R^2^=0.440 / Personal Distress: R^2^=0.270 | | |
| Interpersonal and Social Empathy Index (ISEI) | Segal et al. (2013) | To develop and examine the psychometric properties of ISEI | Cronbach's alphas:  **Total:** α=0.85  **SS-Macro Perspective Taking:** α=0.77  **SS-Cognitive Empathy:** α=0.76  **SS-Self-other Awareness:** α=0.69  **SS-Affective Response:** α=0.64 | EFA:  **ISEI - 4 Factors Model (Macro Perspective Taking, Cognitive Empathy, Self-other Awareness, Affective Response)** = % variance: not informed, Factor loadings > 0.40  CFA:  **ISEI - 1 Factor Model (with the first indicator fixed to set the unit of the factor):** χ²=286.11; CFI=0.91; WRMR=1.11; RMSEA=0.10  **ISEI - Uncorrelated 4 Factors Model (Macro Perspective Taking, Cognitive Empathy, Self-other Awareness, Affective Response):** χ²=1555.10; CFI=0.30; WRMR=4.12; RMSEA=0.26  **ISEI - Correlated 4 Factors Model (Macro Perspective Taking, Cognitive Empathy, Self-other Awareness, Affective Response):** χ²=162.59; CFI=0.96; WRMR=0.78; RMSEA=0.06 | | |
| The Mexican Empathy Scale (MxES) | Mendez et al. (2011) | To translate, adapt and investigate the psychometric properties of MES in the American context. | Cronbach's alphas: Sample 1:  **SS-Empathic Compassion:** α=0.91  **SS-Indifference:** α=0.79  **SS-Cognitive Empathy:** α=0.85  **SS-Disturbance:** α=0.79 | EFA: Sample 1: 4 factors:  **Factor 1: Empathic Compassion** = 21% of variance  **Factor 2: Indifference =** 14% of variance  **Factor 3: Cognitive Empathy =** 5.9% of variance  **Factor 4: Disturbance =** 3.2% of variance  Factor loadings > 0.40 for all solutions  Convergent:  **SS- Empathic Compassion x IRI SS: r**=0.28 to 0.76  **SS-Cognitive Empathy x IRI SS:** r= -0.12 to 0.25  **SS-Indifference x IRI SS:** r= -0.71 to 0.16  **SS-Disturbance x IRI SS:** r=0.01 to 0.65  Predictive: Sample 2:  **High cognitive empathy scores predict** greater **accuracy to detect subtle changes in emotional expression** | | |
| Positive Empathy Scale (PES) | Yue et al. (2016) | To translate and investigate the psychometric properties of PES for the Chinese population. | Cronbach's alphas:  α=0.84  T.R. (4w):  ICC=0.78 | EFA:  **PES - 1 Factor Model (Positive Empathy):**  χ²=36.40; CFI=0.98; TLI=0.95; SRMR=0.03; RMSEA=0.06; Factor loadings > 0.55  CFA:  **PES - 1 Factor Model (Positive Empathy):** χ²=69.55; CFI=0.93; TLI=0.90; SRMR=0.04; RMSEA=0.09; Factor loadings > 0.57  Convergent:  **PES x IRI SS-Empathic Concern:** r=0.31  **PES x Positive and Negative Affect Scale SS-Positive Affect (related construct):** r=0.42  **PES x Prosocial Tendencies Measure (related construct):** r=0.41  **PES x Satisfaction With Life Scale (related construct):** r=0.23  Discriminant:  **PES x IRI SS-Perspective Taking:** r= -0.03  **PES x Perceived Stress Scale-10 (related construct):** r= -0.07 | | |
| Empathic Behavior Scale (ECE) | Auné et al. (2017) | To develop and present the psychometric characteristics of a brief original self-report, the ECE | Cronbach's alphas:  α=0.82 | EFA:  KMO=0.87; Bartlett test of sphericity (χ²=2399.5, p<0.001)  **1 Factor Model** = 48.8% of variance  CFI=0.92; GFI=0.99; RMSR=0.05; factor loadings > 0.56  Convergent:  **ECE x Self-perception of Prosocial Behavior (related construct):** r=0.54  **ECE x Perspective Take-Prosocial Skills Scale (related construct):** r=0.48  **ECE x Self-perception of prosocial behavior SS-Prosociality (related construct):** r=0.28  **ECE x Self-perception of prosocial behavior SS-Giving (related construct):** r=0.34  **ECE x Self-perception of prosocial behavior SS-Altruism (related construct):** r=0.27  **ECE x Big Five Questionnaire (related construct):** r=0.02  Known groups (Gender):  **ECE - women>men:** p<0.000,1, d=0.55 | | |
| Empathy Components Questio-nnaire (ECQ) | Batchelder et al. (2017) | To develop and evaluate the psychometric properties of the ECQ | Cronbach's alphas:  α=0.70 to 0.81 | EFA: Sample 1:  KMO=0.71; Bartlett test of sphericity (1852.64, p<0.001)  **5 Factors Model (Affective Reactivity, Cognitive Drive, Affective Ability, Affective Drive, Cognitive Ability)** = 47.50% of variance; factor loadings > 0.40  CFA: Sample 2:  **ECQ- Model 1:** χ²=754.608; RMSEA=0.07; CFI=0.82; GFI=0.82; AGFI=0.78; SRMR=0.08  **ECQ- Model 2:** χ²=611.28; RMSEA=0.06; CFI=0.86; GFI=0.84; AGFI=0.80; SRMR=0.07  **ECQ- Model 3:** χ²=597.23; RMSEA=0.06; CFI=0.87; GFI=0.84; AGFI=0.81; SRMR=0.07  **ECQ- Model 4:** χ²=502.36; RMSEA=0.05; CFI=0.90; GFI=0.85; AGFI=0.82; SRMR=0.06  Known groups (Gender):  *Sample 1:*  **ECQ total - women=men:** NS  *Sample 2:*  **ECQ total - women>men:** p<0.001  Predictive:  **Stage one**: sex contributed significantly to the regression model: R^2^=0.02.  **Stage two**: introduction of the five empathy components from the ECQ: explained an additional 30% of variation in Social Interest Index-Short Form scores. The Cognitive Ability and Affective Drive subscales are the most significant predictors of SII-SF scores. | | |
| Empathy Gradient Questio-nnaire (EGQ) | Hollar (2017) | To develop and study psychometrics aspects of the EGQ, a scale to assess empathy within the context of psychological distance/relatedness to other individuals and even to other species. | Cronbach’s alphas:  **SS-Family Empathy:** α=0.80  **SS-Friend Empathy:** α=0.86  **SS-Peer Empathy:** α=0.87  **SS-Distant Other Empathy:** α=0.89  **SS-Species Empathy:** α=0.84 | CFA:  **EGQ - 1 Factor Model**: χ²=1475.60; CFI=0.85; GFI=0.96; AGFI=0.96; RMSEA=0.18; SRMR=0.10  **EGQ - 5 Factors Model (Family Empathy, Friend Empathy, Peer Empathy, Distant Other Empathy and Species Empathy):** χ²=961.90; CFI=0.91; GFI=0.98; AGFI=0.98; RMSEA=0.12; SRMR=0.06 (69.8% of variance)  Known groups:  There were no significant effects for age, gender, or race on overall empathy or for each of the five subscales | | |
| Cognitive, Affective,  and Somatic Empathy Scales (CASES) | Park et al. (2019) | To translate and investigate the psychometric properties of the CASES in the Chinese context. | Cronbach’s alphas:  **1 Factor Model:** α=0.89  **2 Factors Model:**  **SS-Positive:** α=0.81  **SS-Negative:** α=0.82  **3 factor model:**  **SS-Affective:** α=0.76  **SS-Cognitive:** α=0.80  **SS-Somatic:** α=0.80 | CFA:  **CASES - 1 Factor Model (Empathy):**  χ²=1411.14; CFI=0.65; SRMR=0.08; RMSEA=0.08  **CASES - 2 Factors Model (Positive and Negative valence):**  χ²=1369.24; CFI=0.67; SRMR=0.08; RMSEA=0.08  **CASES - 3 Factors Model (Affective, Cognitive and Somatic Empathy):**  χ²=1106.86; CFI=0.76; SRMR=0.07; RMSEA=0.07  Convergent:  **CASES x IRI total:** r= 0.48  **CASES x IRI SS:** r= 0.50 to 0.61  **CASES x EQ total:** r= 0.48  **CASES x EQ SS:** r= 0.36 to 0.51  **CASES x Emotion Contagion Scale (related construct):** r= 0.62  **CASES x Emotion Contagion Scale SS (related construct):** r= 0.37 to 0.55 | | |
| Online Empathy Questionnaire (QoE) | Miguel et al. (2018) | To assess the validity of the QoE. | Cronbach’s alphas:  **Total:** α=0.82  **SS-Responsiveness:** α = 0.84  **SS-Respect:** α = 0.69  **SS-Availability:** α = 0.82  T.R. (70d) n=48:  **Mean differences 1ª x 2ª application:**  **Total:** d=0.14  **SS-Responsiveness:** d= -0.01  **SS-Respect:** d=0.25  **SS-Availability:** d=0.10 | Convergent:  **QoE x Personality Factor Battery SS-Socalization (related construct):**  **Factors:** r=0.42 to 0.63; **Total score:** r=0.71  **QoE x Dirty Dozen (D-12) (related construct):**  **Factors:** r= -0.45 to -0.18; **Total score:** r= -0.41  **QoE x Dula Dangerous Driving Index (related construct):**  **Factors**: r= -0.50 to 0.23; **Total score:** r= -0.47  **QoE x Dimensional Clinical Personality Inventory2 SS-Aggressiveness (related construct):**  **Factors**: r= -0.58 to -0.33; **Total score:** r= -0.58  **QoE x (Dimensional Clinical Personality Inventory 2 SS-Isolation (related construct):**  **Factors**: r= -0.41 to -0.24; **Total score**: r= -0.45  **QoE x Brazilian Loneliness Scale (related construct):**  **Factors** r= -0.31 to -0.08; **Total score**: r= -0.22  Discriminant:  **QoE x Reasoning Test Battery 5- Abstract Reasoning:**  **Factors**: r= -0.16 to 0.01; **Total score**: r= -0.11  **QoE x (Reasoning Test Battery 5 - Verbal Reasoning:**  **Factors**: r= -0.01 to 0.00; **Total score**: r=0.00  Known groups (Gender):  **Total score - women>men**: d=0.60  **SS-Responsiveness - women>men:** d=0.72  **SS-Respect - women>men:** d=0.27  **SS-Availability - women>men:** d=0.13 | | |
| Pictorial Empathy Test (PET) | Lindeman et al. (2018) | To develop and validate the PET. | Cronbach’s alphas:  *Sample 1:*  α=0.90  *Sample 2:*  α=0.90  T.R. 7m: Sample 3:  r= 0.77 | CFA: Sample 2:  **PET - 1 Factor Model:** χ²=440.85; CFI=0.95; SRMR=0.03; RMSEA=0.10  **PET - 1 Factor Model (with correlated error):** χ²=45.34; CFI=0.99; SRMR=0.01; RMSEA=0.05; Factor loadings > 0.64  Convergent:  *Sample 2:*  **PET x EQ total:** r=0.48  **PET x EQ SS:** r=0.26 to 0.53  **PET x Symptoms of Autism Spectrum Disorders (related construct):** r= -0.32  **PET x Feminine Sex Role Identity (related construct):** r=0.51  **PET x Intuitive Thinking (related construct):** r=0.26  *Sample 3:*  **PET x Basic Empathy Scale SS:** r=0.26 to 0.46  Discriminant: Sample 2:  **PET x Masculine Sex Role Identity:** r=0.02  **PET x Rational/Experiential Multimodal Inventory SS-Need for Cognition:** r= -0.08  Known groups (Gender): Sample 3:  **PET - women>men:** p<0.001 | | |
| Single Item Trait Empathy Scale (SITES) | Konrath et al. (2018) | To develop and validate the SITES | T.R. (2w) n=367:  r= 0.57  T.R. (3w) n=76:  r= 0.67  T.R. (6m) n=58:  r= 0.62 | Kurtosis=-0.80 to 1.90/ Skewness=-1.15 to 0.29  Convergent:  **SITES x IRI SS: r**=-0.08 to 0.50  **SITES x Single Item Narcissism Scale (related construct):** r= -0.32 to -0.08  **SITES x Narcissistic Personality Inventory-40 (related construct):** r= -0.16  **SITES x Satisfaction with Life Scale (related construct): r**=0.10  **SITES x Epidemiologic Studies Depression Scale (related construct):** r= -0.07  **SITES x State-Trait Anxiety Index (related construct):** r= -0.08  Known groups (Gender):  **women>men:** d=0.48 | | |
| ACME-CE =Affective and Cognitive Measure of Empathy - Cognitive Empathy; AGFI =The adjusted goodness of fit index; AIC=Akaike’s Information Criterion; CAIC =Bozdogan’s consistent version of the AIC; CE =Cognitive empathy; CFA =Confirmatory Factor Analysis; CFI =Comparative Fit Index; EFA =Exploratory Factor Analysis; ESEM =Exploratory Structural Equation Model; GFI =Goodness of Fit Index; ICC =Intraclass correlation; IFI =Incremental Fit Index; IRI–EC =total score for the IRI’s empathic concern items; IRI–FS =total score for the IRI’s fantasy items; IRI–PD =total score for the IRI’s personal distress items; IRI–PT =total score for the IRI’s perspective-taking items; KMO = The Kaiser-Meyer-Olkin; MLχ2 =Maximum Likelihood; NNFI =Non-Normed Fit Index; NS =Not Significant; r =Correlation Coefficient; R²=Determination Coefficient; RMR =Root Mean Square Residual; RMSEA =Root Mean Square Error of Approximation; SBχ2 = Satorra–Bentler adjustments; SS =Subscale; SRMR =Standardized Root Mean Square Residual; TLI =Tucker-Lewis Index; T.R. =Test Retest; WLSMV =Weighted least squares; WRMR =Weighted Root Mean Square Wesidual; X² =Chi Square; α =Cronbach’s alpha | | | | |  |  |

**Supplementary Material 3**

SM3.1. Forest plot for the reliability generalization (Cronbach’s alpha) and Funnel plot for the assessment of publication bias for ***Empathy Quotient***

| 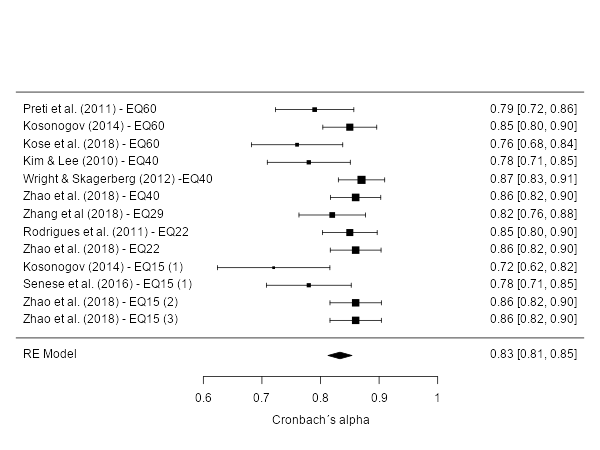 | 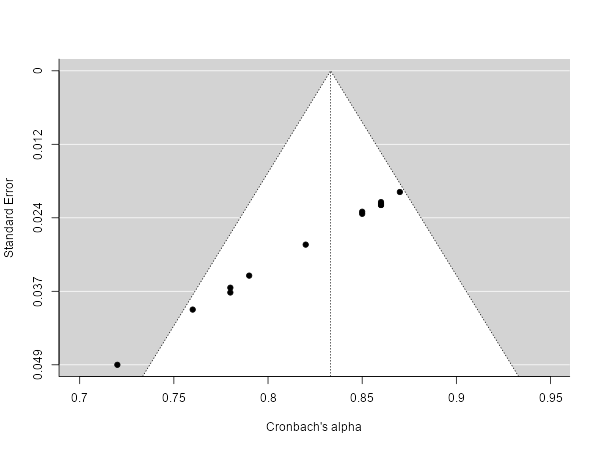 |
| --- | --- |

*(1) Modelo de Muncer & Ling, 2006; (2) Modelo de Guan et al., 2012; (3)* *Modelo de Guan et al., 2012 - modificado*

SM3.2. Forest plot for the reliability measure (test-retest) and Funnel plot for the assessment of publication bias for ***Empathy Quotient***

| 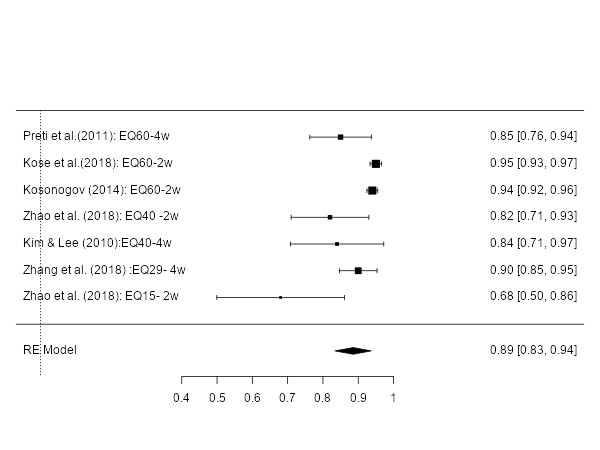 | 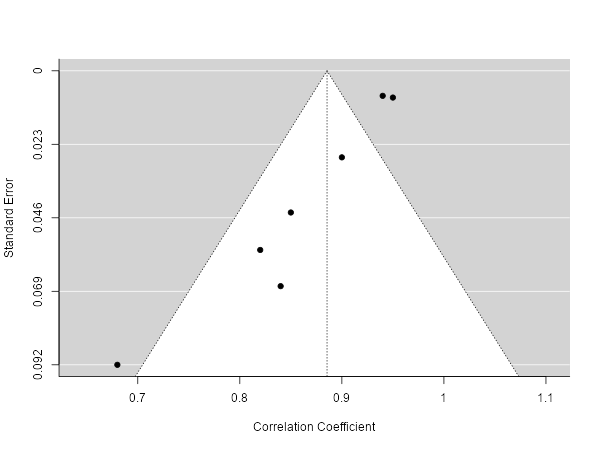 |
| --- | --- |

SM3.3. Forest plot for the convergente measure and Funnel plot for the assessment of publication bias for ***Empathy Quotient***

| 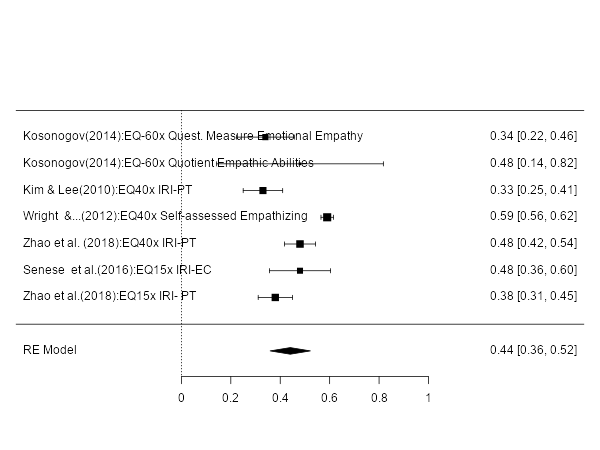 | 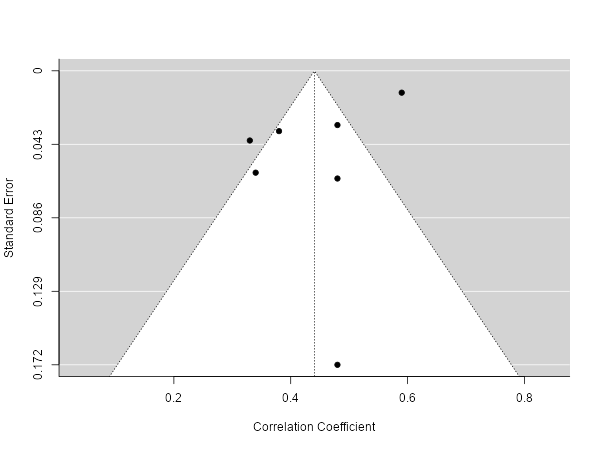 |
| --- | --- |
| 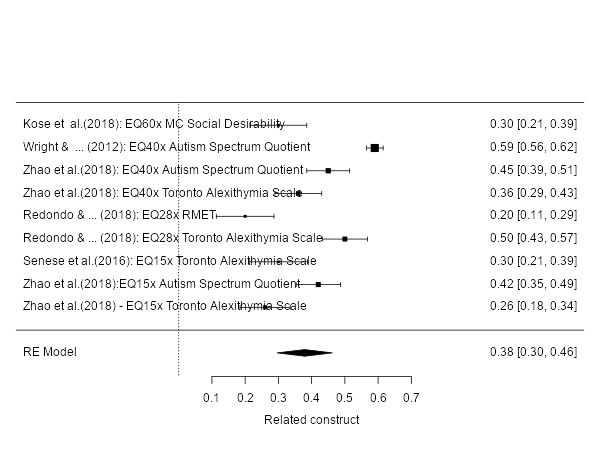 | 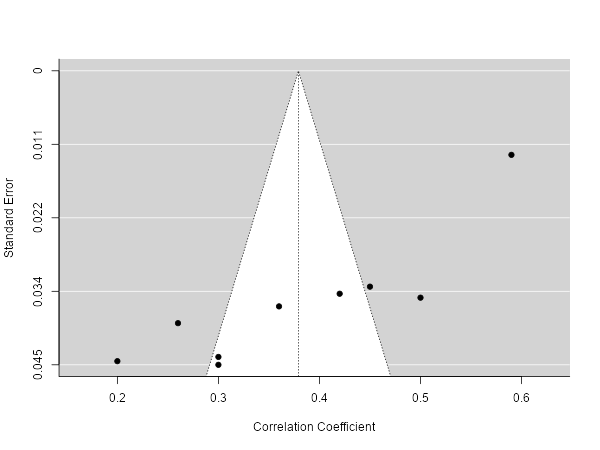 |

**Supplementary Material 4**

SM4.1. Forest plot for the reliability generalization (Cronbach’s alpha) and Funnel plot for the assessment of publication bias for ***Interpersonal Reactivity Index Subscales***

| 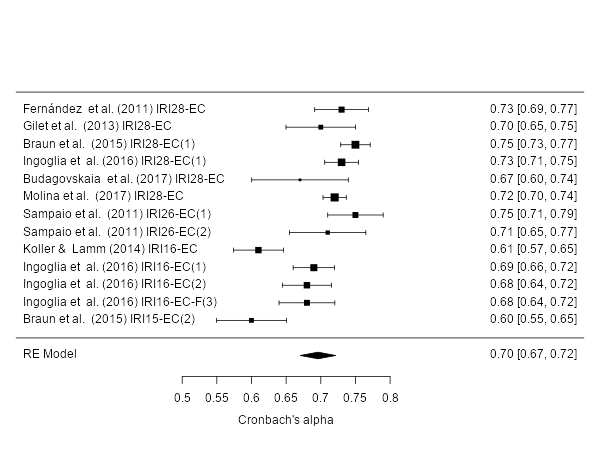 | 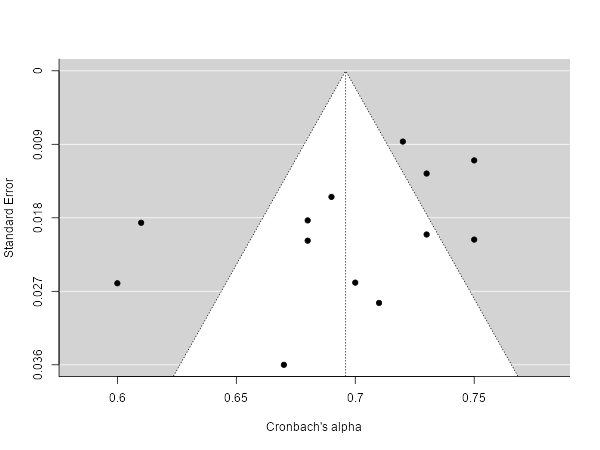 |
| --- | --- |
| 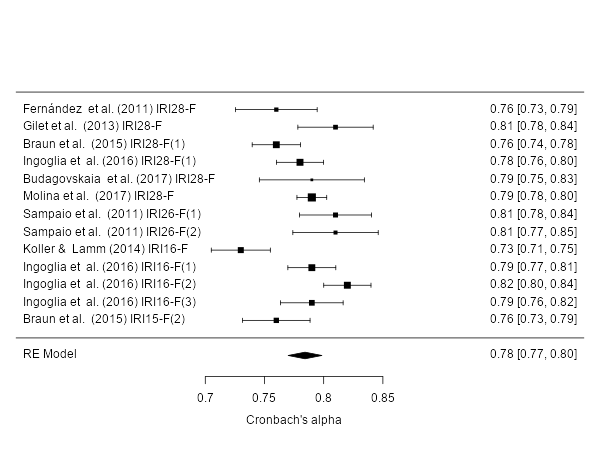 | 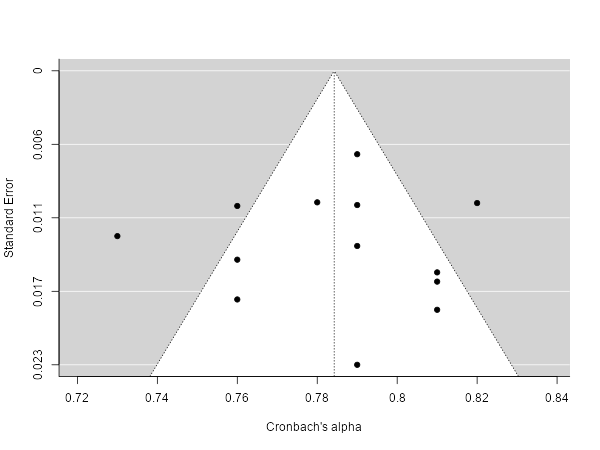 |
| 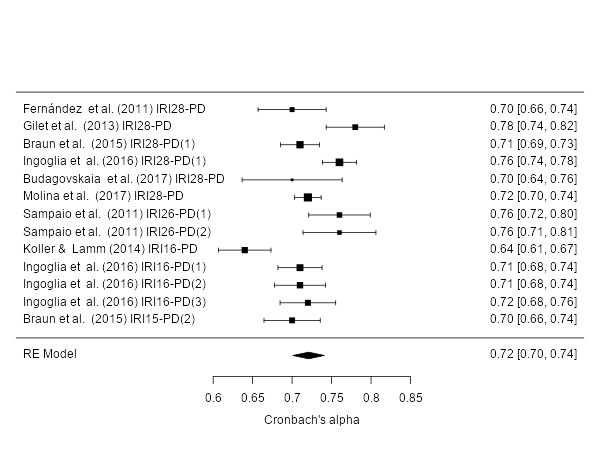 | 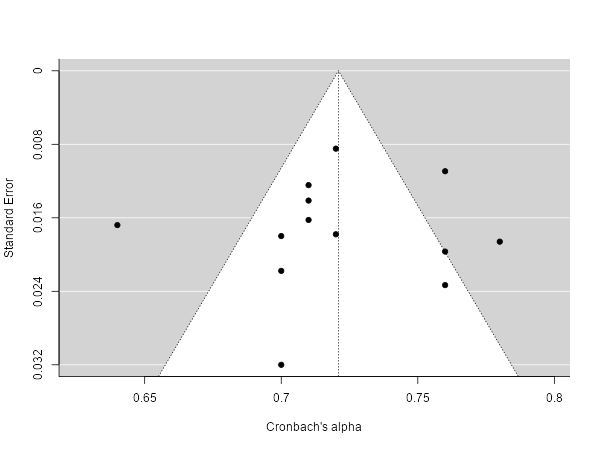 |
| 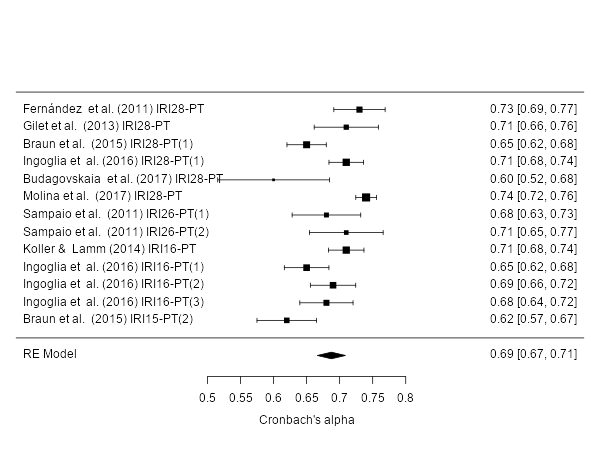 | 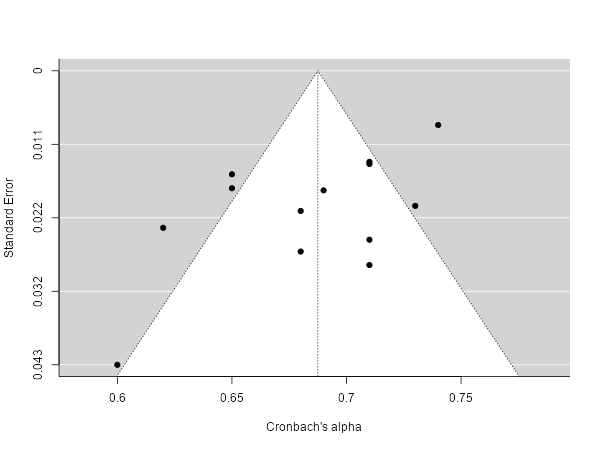 |

EC: Empathic Concern; F: Fantasy; PD: Personal Distress; PT: Perspective Taking; (1) Sample 1; (2) Sample 2; (3) Sample 3

SM4.2. Forest plot for the convergente measure and Funnel plot for the assessment of publication bias for ***Interpersonal Reactivity Index***

| 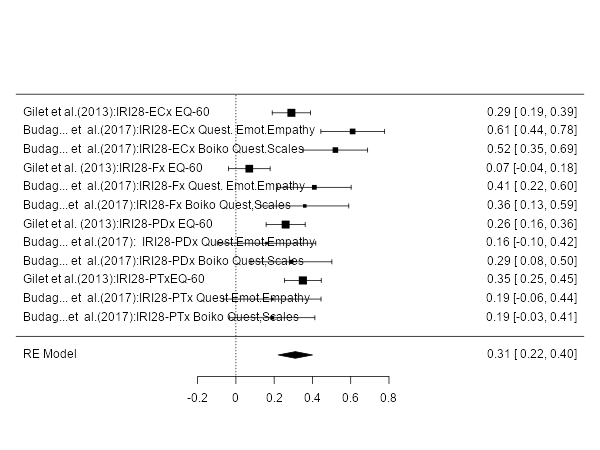 | 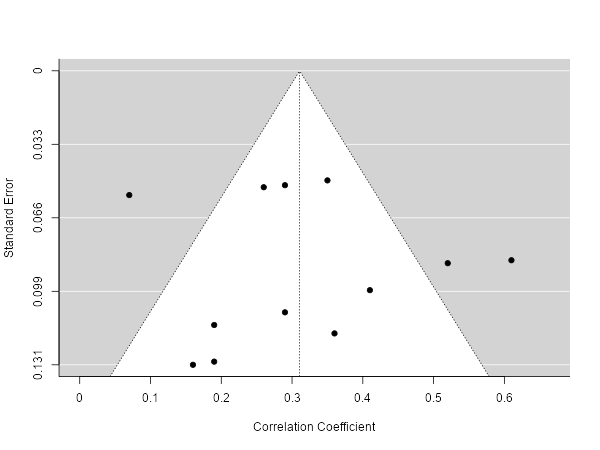 |
| --- | --- |
| 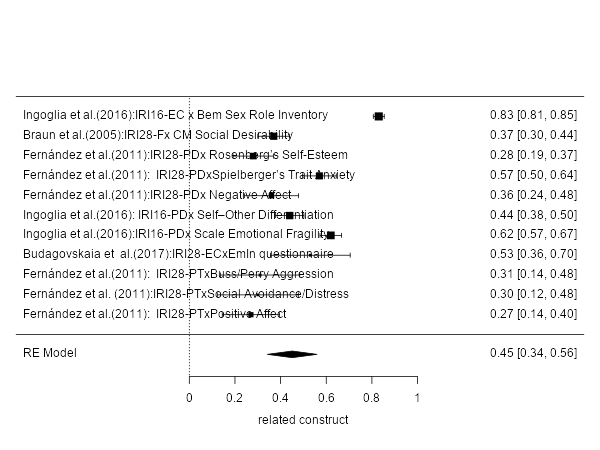 | 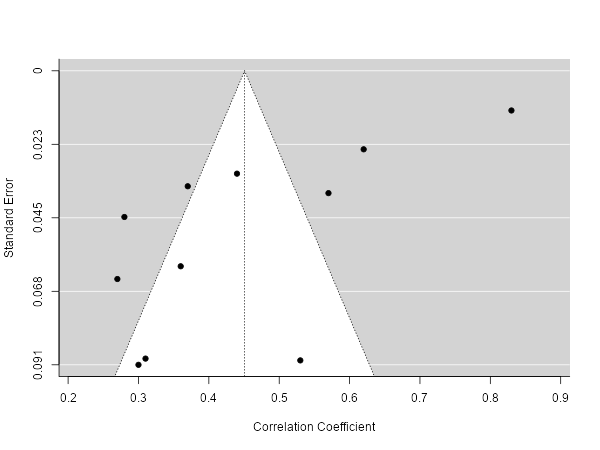 |

**Supplementary Material 5**

SM5.1. Forest plot for the reliability generalization (Cronbach’s alpha) and Funnel plot for the assessment of publication bias for **Questionnaire of Cognitive and Affective Empathy**

| 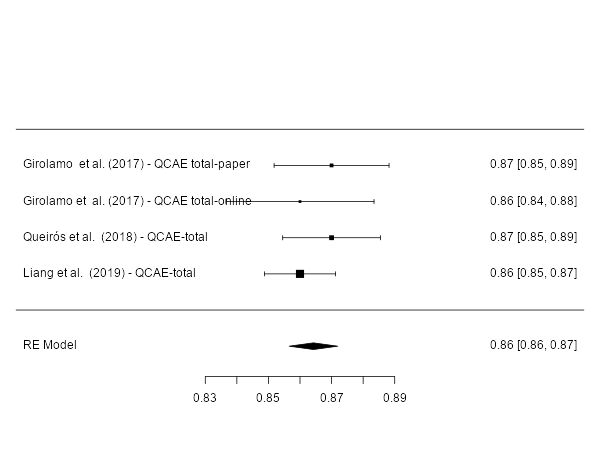 | 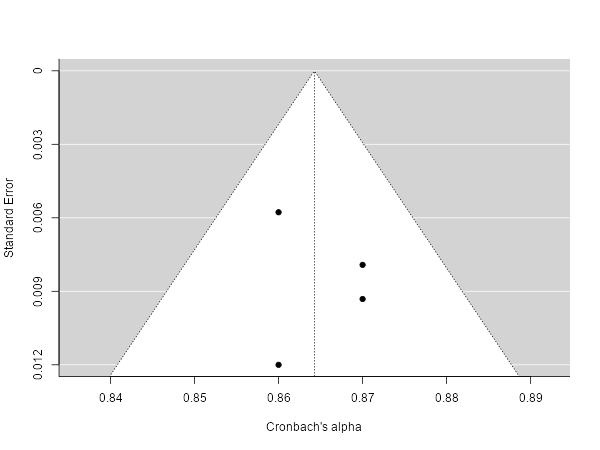 |
| --- | --- |
| 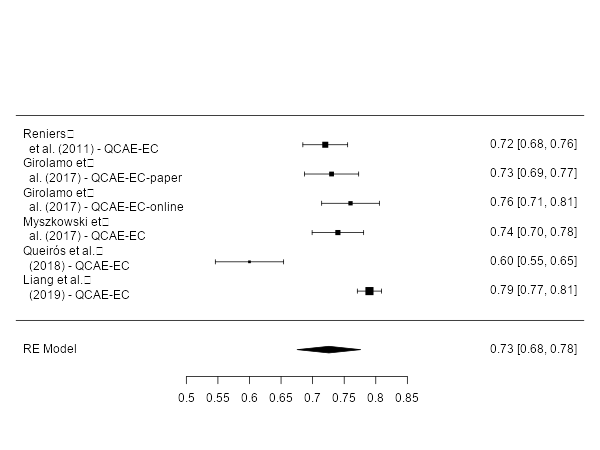 | 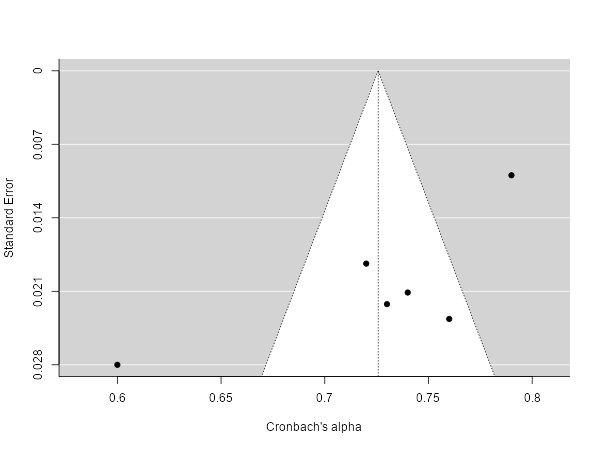 |
| 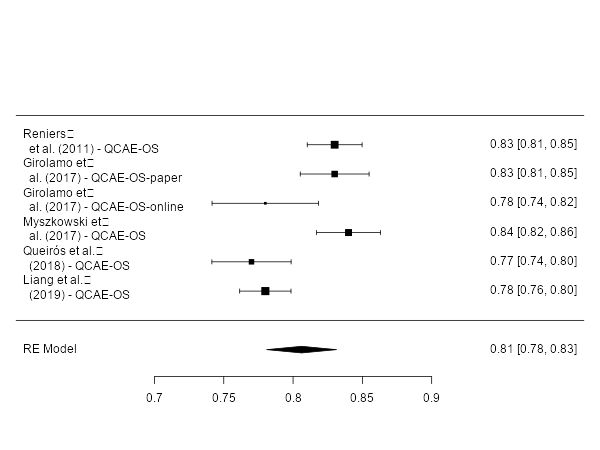 | 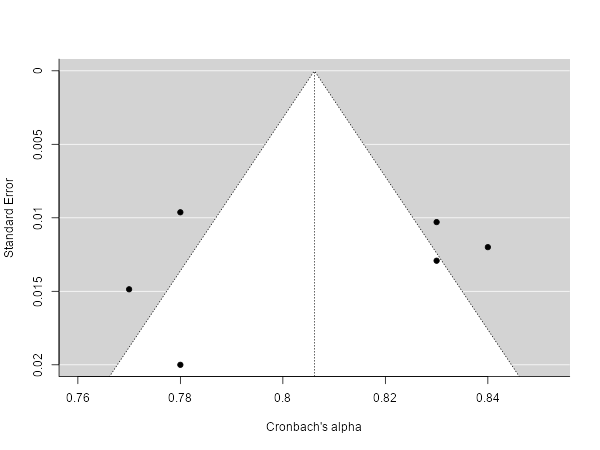 |
| 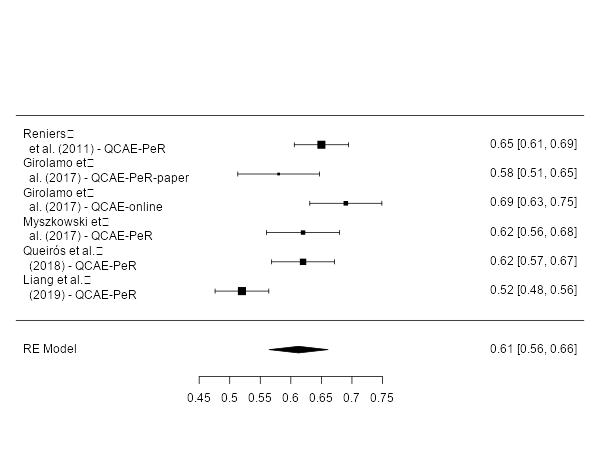 | 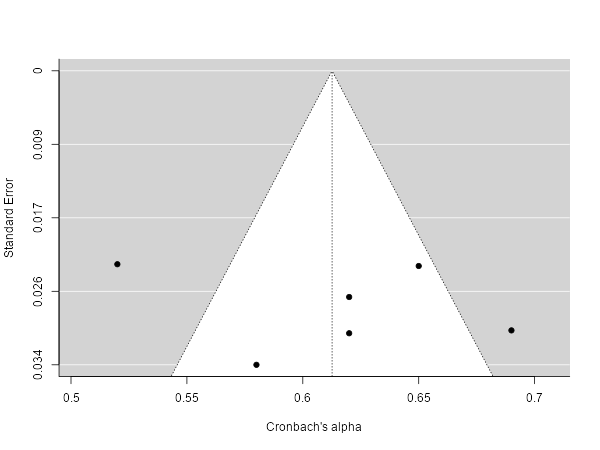 |
| 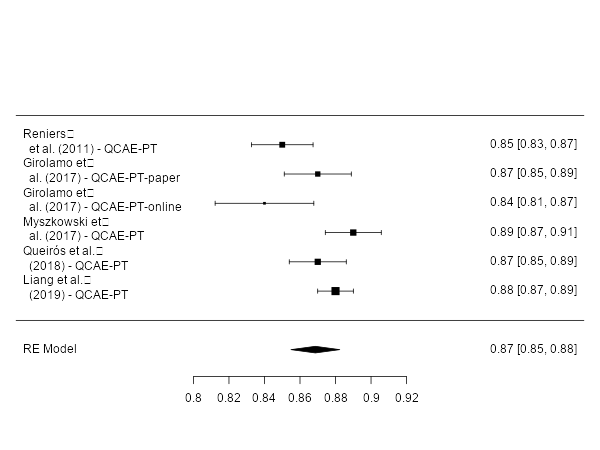 | 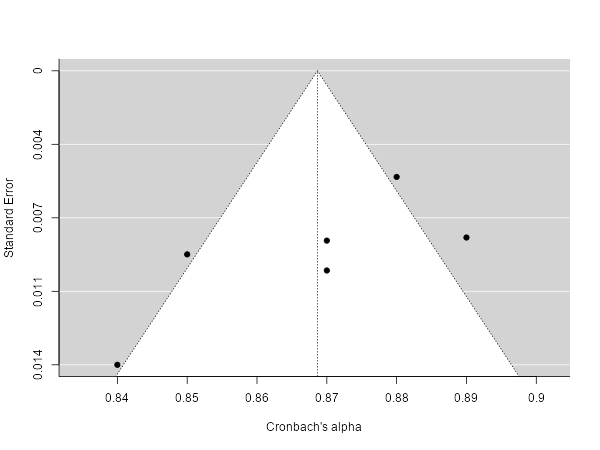 |
| 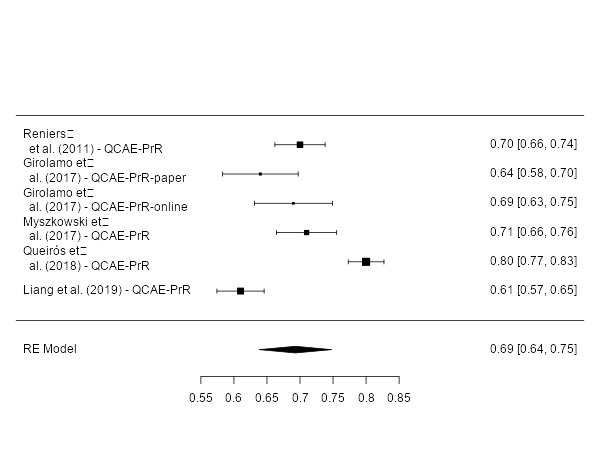 | 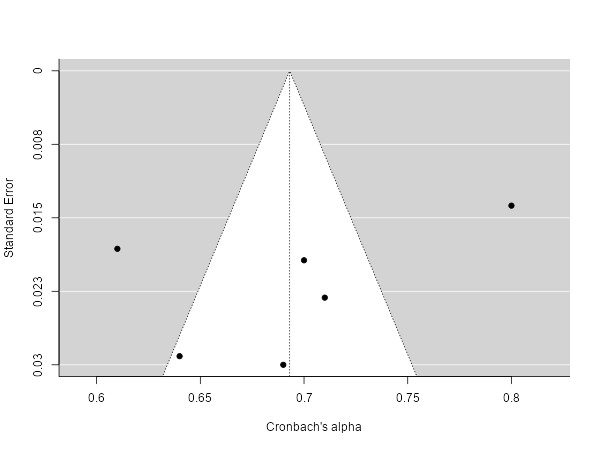 |

EC: Emotion Contagion; OS: Online simulation; PeR: Peripheral Responsivity; PT: Perspective Talking; PrP: Proximal Responsivity

SM5.2. Forest plot for the convergente measure and Funnel plot for the assessment of publication bias for **Questionnaire of Cognitive and Affective Empathy**

| 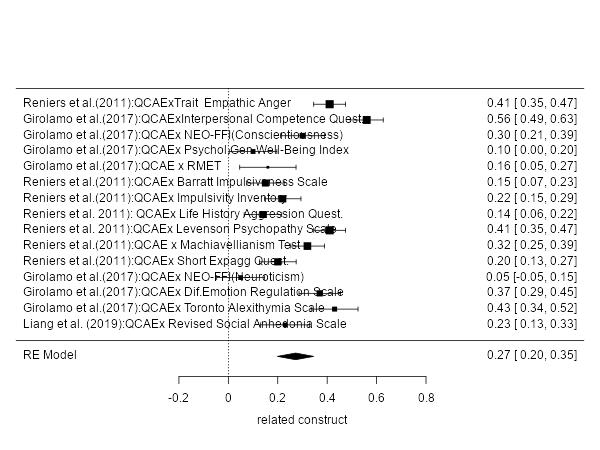 | 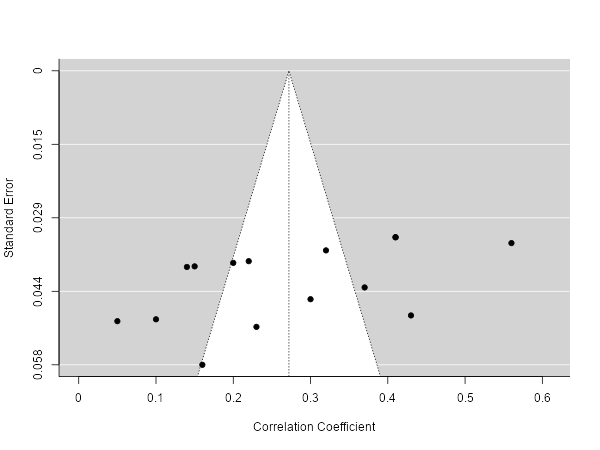 |
| --- | --- |
